# Intravenous Lipid Emulsion Attenuates Hepatic, Renal, Cerebral and Testicular Histopathological Injury but Not Renal Dysfunction or Cardiac Histological Change in an Acute Haloperidol Overdose Model in Rats: Implications for Veterinary Toxicology

**DOI:** 10.3390/vetsci13090890

**Published:** 2026-08-31

**Authors:** Muhammet Can, Cihangir Işık, Gülay Turan, Merve Yılmaz, Sermin Biçer, Mustafa Hilmi Yaranoğlu

**Affiliations:** 1 Department of Forensic Medicine, Faculty of Medicine, Balıkesir University, 10145 Balıkesir, Türkiye; 2 Department of Forensic Medicine, Balıkesir Atatürk City Hospital, 10100 Balıkesir, Türkiye; 3 Department of Pathology, Faculty of Medicine, Balıkesir University, 10145 Balıkesir, Türkiye; 4 Department of Biochemistry, Faculty of Medicine, Balıkesir University, 10145 Balıkesir, Türkiye; 5 Department of Pharmacology, Faculty of Pharmacy, Istanbul Yeni Yuzyil University, 34010 Istanbul, Türkiye; 6 Experimental Animal Production, Care and Research Centre, Balıkesir University, 10145 Balıkesir, Türkiye

**Keywords:** haloperidol, butyrophenone neuroleptic, veterinary toxicology, intravenous lipid emulsion, lipid rescue therapy, comparative medicine, multi-organ histopathology, laboratory animal model, Wistar rat, spermatogenesis

## Abstract

**Background:** Butyrophenone neuroleptics are used in veterinary practice—haloperidol in the capture and translocation of wild ungulates, azaperone in swine—and antipsychotics prescribed to owners are a recognised cause of accidental toxicosis in dogs and cats. Organ-level toxicity data for this drug class are nonetheless almost absent in any species. Intravenous lipid emulsion (ILE) is an established antidote in small-animal emergency medicine for lipophilic intoxications, but the veterinary evidence base consists of case reports with clinical endpoints, and no controlled study has examined what ILE does to the tissues themselves. This study used an acute rodent overdose model to provide that organ-level assessment. **Methods:** Forty male Wistar albino rats were randomised to four groups of ten: control (saline 2 mL/kg oral gavage), HP (17.5 mg/kg oral gavage), HP + ILE (17.5 mg/kg HP plus 20% ILE 7.5 mL/kg intravenously at 0.5 h and 8 h, an empirical regimen) and ILE alone. Endpoints were assessed at 24 h. Seventeen histopathological parameters across seven organs were scored semi-quantitatively by a blinded pathologist (n = 10 per group), with independent re-scoring of a stratified random 30% subsample by a second blinded pathologist. Serum total oxidant status (TOS), total antioxidant status (TAS), oxidative stress index (OSI) and routine biochemistry were measured in animals with adequate serum (n = 34 and n = 35). Comparisons used Kruskal–Wallis and Mann–Whitney U tests with Benjamini–Hochberg false discovery rate (FDR) control. **Results:** Haloperidol produced significant injury in the liver, kidney, brain and testis (all *p* < 0.001 vs. control) and mild histological cardiac changes (degeneration *p* = 0.014; congestion *p* = 0.034); lung and eye were unaffected. Serum urea was markedly elevated in HP (67.4 ± 13.2 vs. 46.5 ± 8.8 mg/dL; *p* = 0.001, *d* = 1.82) and HP + ILE (61.5 ± 8.7 mg/dL; *p* = 0.003), with unchanged creatinine, and correlated strongly with renal histopathology (renal inflammation vs. urea ρ = 0.74, *p* < 0.001). ILE significantly attenuated histopathological injury in 8 of the 12 haloperidol-associated parameters after FDR correction (renal congestion *q* = 0.032, *r* = 0.70; testicular Johnsen score *q* = 0.032, *r* = 0.66); the testicular score improved significantly relative to HP (*q* = 0.032) but remained statistically indistinguishable from control (*p* = 0.072), which does not establish equivalence. ILE did not reduce serum urea (HP + ILE vs. HP, *p* = 0.26) and did not modify the mild histological cardiac changes (*p* ≥ 0.40); no functional or electrophysiological cardiac endpoint was assessed. No group difference was detected in serum TOS or OSI (*p* = 0.43 and *p* = 0.51), whereas TAS was significantly reduced in the ILE-alone group (*p* = 0.011); the biological interpretation of this TAS finding remains uncertain, because interference of residual lipaemia with the spectrophotometric assay could not be excluded and would also propagate to the derived OSI. No tissue oxidative marker was measured. Transaminases were not elevated by haloperidol and showed no correlation with hepatic histopathology (|ρ| ≤ 0.17, all *p* > 0.34). ILE alone caused no histopathological abnormality at this dose and time point. Inter-observer agreement was high (95.6% exact agreement; median weighted κ = 0.893). **Conclusions:** In this acute overdose model, ILE is associated with a differential and structurally incomplete histopathological response: microscopic injury improves in four organs while renal function does not, and the mild histological cardiac changes are unaltered. Serum transaminases did not reflect concurrent organ-level injury at 24 h. Serum TOS and OSI showed no detectable group differences, while TAS was reduced in the ILE-alone group with an uncertain biological interpretation. Given the analytical uncertainty and the limited sample size, these indices provided no usable surrogate information about organ-level injury in this model. These findings define which organ systems respond and require confirmation in target veterinary species before informing clinical practice.

## Simple Summary

Haloperidol belongs to the butyrophenone group of tranquillisers, a class that veterinarians use directly: haloperidol itself is given to wild antelope, deer and zebra during capture and translocation, and its relative azaperone is licensed for pigs. Dogs and cats are also poisoned by swallowing their owners’ antipsychotic tablets. Despite this, almost nothing is known about what a toxic dose of a butyrophenone does to the internal organs of any animal. Intravenous lipid emulsion—a fat-containing fluid given into a vein—is already a routine antidote in small-animal emergency practice for fat-soluble poisons such as permethrin and ivermectin, but its use rests on case reports that describe how animals behaved, not on any examination of their tissues. To provide that missing tissue-level information, we built a deliberately extreme laboratory model: forty rats received a single very high dose of haloperidol, far above any dose a veterinarian would give, with or without lipid emulsion, and seventeen microscopic tissue features across seven organs were examined at 24 h together with blood tests. Haloperidol damaged the liver, kidney, brain and testis, raised blood urea, and caused mild microscopic heart changes. Lipid emulsion significantly reduced the microscopic damage in eight of the twelve affected measures, covering liver, kidney, brain and testis, and the testicular measure showed the largest improvement. It did not correct the raised blood urea and did not change the mild heart findings, although the heart was examined only under the microscope and not by electrical or blood-pressure monitoring, so a functional heart benefit remains untested. Blood liver enzymes stayed normal even though the liver was clearly damaged—a warning for clinicians who rely on such tests in poisoned patients. The blood markers of oxidative stress gave no clear signal either: two of the three did not differ between groups, while the third changed only in the lipid-only group, possibly because fat in the sample interfered with the laboratory measurement rather than because of a real biological effect. Lipid emulsion given on its own caused no microscopic tissue injury in any organ at this dose and at 24 h, although complications known to occur in treated animals, such as high blood fat levels and pancreatitis, were not assessed. This is an acute rodent overdose model; it maps which organs respond and which do not, and generates questions for veterinary study rather than recommendations for treating animal patients.

## 1. Introduction

Butyrophenone neuroleptics are not solely human drugs. Haloperidol has been used for four decades as a short- to medium-acting neuroleptic in wildlife capture and translocation, where it suppresses the alarm reaction and improves tractability in red hartebeest, blesbok, springbok, duiker, impala and zebra [1]. It is administered to roe deer to attenuate the physiological stress of drive-net capture [2], and sustained-release formulations are incorporated into tranquilliser protocols for large ungulates during transport and holding [3]. Its close structural relative azaperone is licensed for swine. Adverse effects in these veterinary applications are recognised but poorly characterised: extrapyramidal reactions have been reported in several treated wild species, and paradoxical excitement at higher doses in larger ungulates has led practitioners to reduce doses empirically [1,3]. What a supratherapeutic butyrophenone exposure does to the internal organs of any species has, to our knowledge, never been systematically described.

A second route of veterinary exposure is accidental. Human pharmaceuticals account for roughly a third of reported companion animal poisonings, most often through ingestion of medication stored within the household, and psychotropic agents including antipsychotics form an established category within this group [4,5]. Dogs and cats generally require lower doses than humans and are correspondingly sensitive to overdose. In human medicine, haloperidol has been in clinical use for more than six decades, and its overdose syndrome—extrapyramidal signs, catalepsy, hypothermia, anticholinergic effects and cardiac conduction disturbance including QTc prolongation that may progress to torsades de pointes—is well described [6,7]. No specific antidote exists in either human or veterinary practice, and management is supportive [4].

Intravenous lipid emulsion (ILE) has become a routine element of small-animal emergency toxicology. It is used in dogs and cats for permethrin, macrocyclic lactone, baclofen, non-steroidal anti-inflammatory and local anaesthetic intoxications, and reviews of veterinary practice now treat it as an established, if incompletely evidenced, antidote [8,9]. Controlled veterinary data exist for permethrin toxicosis in cats [10], and larger case series describe its use in ivermectin-overdosed cats [11]. The parallel human literature followed the same trajectory, beginning with local anaesthetic systemic toxicity and extending to other lipophilic agents [12,13,14]. The dominant mechanistic account, the “lipid sink” model, proposes that an expanded intravascular lipid phase sequesters lipophilic xenobiotics away from target tissues and lowers their effective receptor-site concentration [15]; metabolic cardioprotection through fatty-acid substrate provision has also been proposed. Haloperidol is lipophilic and distributes widely into tissue, making it a pharmacologically plausible candidate for lipid rescue.

The veterinary evidence for ILE has an important structural weakness. It rests almost entirely on case reports and case series in which the endpoints are clinical and behavioural—recovery of consciousness, resolution of tremor, survival to discharge. Whether ILE alters what is happening in the tissues, and whether it does so uniformly across organs, has not been established in any species. This matters directly for practice, because ILE is often administered empirically to a collapsing patient without knowing which organs, if any, stand to benefit.

Direct evidence in haloperidol toxicity specifically is sparse and inconsistent. Awad et al. [16] described resolution of rigidity and altered mental status in a single human patient after ILE. Ghaleb et al. [17] reported biochemical and histopathological improvement in the liver and kidney of haloperidol-poisoned rats treated with 10 mL/kg ILE. In contrast, Moshiri et al. [18] found dose- and endpoint-dependent effects in rabbits, with the optimal dose for neurological endpoints (12 mL/kg) differing substantially from that for cardiac endpoints (6 mL/kg). The emerging picture is of an intervention whose efficacy may vary systematically by target organ—a possibility that cannot be resolved by studies examining one or two organs at a time.

Experimentally, haloperidol generates reactive oxygen species, promotes lipid peroxidation and impairs mitochondrial function in neuronal and hepatic tissue [19,20,21], and animal studies have documented hepatocellular injury, renal congestion, impaired spermatogenesis and neuroinflammation [22,23,24]. These organs have, however, been examined in separate studies with heterogeneous dosing regimens, and cerebral, ocular and cardiac tissue has not previously been assessed alongside the classical target organs within a single experiment. It is also unclear whether the serum markers on which clinicians actually rely—transaminases, urea, creatinine and oxidative stress indices—track organ-level injury closely enough to guide management, an assumption made implicitly by much of the existing veterinary and human literature.

The rat was selected as a laboratory model because it permits controlled dosing, complete organ harvest and blinded histopathological scoring that cannot be obtained from clinical veterinary casework, where exposure dose is usually unknown, and post-mortem material is rarely available from survivors. The present study was designed to address three questions: (i) to characterise the histopathological profile of acute high-dose haloperidol toxicity across seven organ systems simultaneously, together with oxidative and biochemical indices; (ii) to determine, organ by organ, whether ILE co-administration attenuates this injury; and (iii) to test whether serum oxidative stress and organ-function markers correspond to the histopathological findings in the same animals. A subsidiary aim, of direct relevance to veterinary practice, was to assess whether ILE administered alone produces any detectable organ injury.

## 2. Materials and Methods

### 2.1. Ethics and Regulatory Compliance

The study was approved by the Experimental Animal Ethics Committee of Balıkesir University, Türkiye (reference 2022/8-2; 27 October 2022) and conducted in accordance with the Turkish Regulation on the Welfare and Protection of Animals Used for Experimental and Other Scientific Purposes (Official Gazette 28141), Directive 2010/63/EU, and the NIH Guide for the Care and Use of Laboratory Animals. Reporting follows the ARRIVE 2.0 guidelines. Animals were supplied by the Balıkesir University Experimental Animal Production, Care and Research Centre.

### 2.2. Animals and Housing

Forty healthy male Wistar albino rats (7–8 weeks old, 250–300 g) were housed under controlled conditions (20–25 °C, 50–60% relative humidity, 12 h light/dark cycle) with *ad libitum* access to standard rat chow (Korkutelim Food Company, Antalya, Türkiye; 23.5% crude protein, 3.3% ether extract, 6.1% crude fibre, 2800 kcal/kg metabolisable energy) and filtered tap water, following seven days of acclimatisation.

Animals were housed in groups in standard polycarbonate cages with softwood bedding. No environmental enrichment was provided.

Only male animals were used to avoid oestrous-cycle-dependent variation in oxidative stress parameters and drug metabolism and to permit assessment of testicular endpoints.

Study design, experimental unit and sample size. The experimental unit was the individual animal. Forty animals were allocated to four groups of ten. The group size was determined by the number of animals authorised by the institutional ethics committee; no a priori statistical power calculation was performed. The primary outcome measures were the ordinal histopathological scores; serum biochemical and oxidative stress indices were secondary outcomes. A sensitivity analysis indicating the effect sizes detectable at the sample sizes obtained is reported in Section 3.2.

Randomisation and control of confounders. Rats were allocated to the four groups by drawing lots, and group mean body weights were checked for balance after allocation. Potential confounders were not controlled: cage position on the rack was not randomised, and the order in which animals were dosed, sampled and processed was neither randomised nor held constant. The implications of this for the interpretation of sample attrition are considered in Section 4.7.

Blinding. The pathologist performing the primary histopathological assessment and the second pathologist performing the reliability re-scoring were both blinded to group allocation (Section 2.8 and Section 2.9). Biochemical and oxidative stress assays were performed by laboratory personnel who were not informed of the group allocation of individual samples. Personnel administering the test substances were necessarily aware of group allocation, since the interventions differ in route and appearance. Statistical analysis was performed on group-labelled data and was therefore not blinded.

Inclusion and exclusion criteria. No a priori inclusion or exclusion criteria were defined for animals. All forty animals completed the protocol, no mortality occurred, and all forty contributed histopathological data. Exclusions applied at the analysis stage are limited to those described in Section 2.5 and Section 2.6 and are reported in full there.

### 2.3. Test Substances and Experimental Groups

Agents used were physiological saline (0.9% NaCl; Polifleks, Polifarma İlaç San. ve Tic. A.Ş., Tekirdağ, Türkiye); haloperidol (NORODOL, 10 mg/2 mL ampoule; Ali Raif Pharmaceutical Industry, Istanbul, Türkiye); and 20% lipid emulsion (Clinoleic 20%, 500 mL; Baxter, Lessines, Belgium; 80% refined olive oil/20% refined soybean oil with purified egg phospholipids, glycerol, sodium oleate and sodium hydroxide).

1. Group 1—Control (n = 10): single oral gavage of saline, 2 mL/kg.
2. Group 2—HP (n = 10): single oral gavage of haloperidol, 17.5 mg/kg.
3. Group 3—HP + ILE (n = 10): single oral gavage of haloperidol, 17.5 mg/kg, followed by 20% ILE 7.5 mL/kg by tail-vein injection at 0.5 h and 8 h.
4. Group 4—ILE (n = 10): 20% ILE 7.5 mL/kg by tail-vein injection at 0.5 h and 8 h.

The haloperidol dose of 17.5 mg/kg was chosen to produce an overt but sublethal acute toxic syndrome, consistent with previously published rodent models [17,22]. The ILE regimen (7.5 mL/kg at 0.5 h and 8 h) was empirical. The dose lies within the range associated with an antidotal effect in prior rodent and rabbit studies [18,25], and the two time points were intended to bracket the period of rising and sustained systemic haloperidol exposure after oral administration: the first infusion shortly after gavage, before absorption is complete, and the second within the interval over which tissue concentrations were expected to remain elevated. No pharmacokinetic measurements were made in this study, and toxicokinetic data for oral haloperidol in the rat at this dose are not available, so the schedule was not anchored to a measured absorption or distribution profile. Because haloperidol has a large volume of distribution, an intravascular lipid phase established 30 min after oral dosing may act on only part of the absorbed drug, and a proportion may already have partitioned into tissue before the first infusion. The consequence for interpretation is explicit: this study cannot determine whether the differential pattern observed reflects an optimal, a suboptimal or a mistimed ILE regimen, and the effects reported are those achievable with this particular schedule rather than the maximum achievable with lipid rescue. Animals were observed at 1, 2, 4, 8, 12 and 24 h after dosing for clinical signs, including posture, locomotor activity, respiratory pattern, response to handling and evidence of pain or distress.

Humane endpoints and adverse events. No humane endpoints were formally defined in the approved protocol, and this is stated here explicitly in accordance with ARRIVE item 16c. Animals were nonetheless monitored at 1, 2, 4, 8, 12 and 24 h, and the study team was instructed to euthanise immediately, by the method described in Section 2.4, any animal showing respiratory compromise, persistent unresponsive recumbency or other signs of severe distress. No animal met these criteria, no animal died before the scheduled endpoint, and no unexpected adverse events occurred. Observed clinical signs are reported in Section 3.1. The absence of pre-specified humane endpoints is acknowledged as a limitation in Section 4.7.

### 2.4. Terminal Procedures and Sample Collection

At 24 h after dosing, animals were killed by decapitation using a dedicated rodent guillotine, without prior administration of injectable or inhalational anaesthetic agents.

This method was selected deliberately and on scientific grounds. The primary endpoints of this study were serum oxidative stress indices and hepatic, renal and cardiac biochemical markers. Injectable anaesthetics used for terminal procedures in rodents—in particular ketamine–xylazine combinations—alter systemic redox balance, glucose homeostasis, hepatic and renal perfusion and serum enzyme activities, and inhalational agents produce comparable confounding, so their use would have compromised precisely the measurements the study was designed to make. In the AVMA Guidelines for the Euthanasia of Animals (2020 edition), decapitation of small laboratory rodents without prior anaesthesia is classified as *acceptable with conditions*. The stated conditions are that the method is used only where its scientific necessity has been established and approved by the relevant animal care and use committee, that personnel are trained and demonstrate proficiency in the technique, and that equipment is properly maintained and kept sharp. All three conditions were met and are documented below. Decapitation is likewise included among the methods permitted for rodents in Annex IV of Directive 2010/63/EU.

The conditions attaching to this method were observed and documented. The procedure was performed by a single operator experienced in the technique; the guillotine blade was inspected and verified as sharp before each session; animals were restrained and handled so as to minimise pre-procedural stress and were not exposed to the procedure being performed on other animals; and decapitation was executed as a single uninterrupted action, in which loss of consciousness is effectively immediate. Decapitation without prior anaesthesia was specifically approved by the institutional ethics committee (approval 2022/8-2) on the scientific grounds described above.

Trunk blood was collected immediately into gel-separator tubes, allowed to clot at room temperature for 30 min and centrifuged at 2000× *g* for 15 min. Serum aliquots were stored at −80 °C until analysis. Tissues were harvested immediately after exsanguination.

### 2.5. Missing Data and Analysable Sample Sizes

Histopathological scores were obtained from all 40 animals. In some animals the trunk blood volume obtained was insufficient for the complete analytical panel, and a small number of samples clotted before separation; consequently, the number of animals contributing biochemical data was lower than 40 and differed between assay panels. Routine biochemistry was available for 35 animals (control n = 8, HP n = 10, HP + ILE n = 10, ILE n = 7) and oxidative stress indices for 34 animals (control n = 7, HP n = 10, HP + ILE n = 10, ILE n = 7). Analysable sample sizes are stated for every endpoint throughout and are summarised in Table 1. No animal or sample was excluded on the basis of its result. The seven ILE-group animals contributing biochemical data are the same seven animals that contributed oxidative stress data, confirmed against the laboratory records.

Sample loss was confined entirely to the two groups that did not receive haloperidol. All twenty animals in the HP and HP + ILE groups yielded complete biochemical and oxidative stress data, whereas five of twenty control and ILE animals lacked routine biochemistry and six of twenty lacked oxidative stress indices. The difference is statistically detectable (Fisher’s exact test, *p* = 0.047 for biochemistry and *p* = 0.020 for oxidative stress). The implications for bias are considered in Section 4.7.

In each of the control and ILE groups, two animals yielded insufficient trunk blood for any analysis and one sample clotted before separation. In the control group the clotted sample nonetheless permitted routine biochemistry but not the oxidative stress panel, which accounts for the difference between the two columns.

For LDH, AST and CK-MB, a further four samples were flagged as haemolysed by the pre-specified criterion in Section 2.6; these were not discarded but analysed under four alternative handling strategies (Section 3.3).

### 2.6. Biochemical Analysis

Aspartate aminotransferase (AST), alanine aminotransferase (ALT), alkaline phosphatase (ALP), lactate dehydrogenase (LDH), urea, creatinine and creatine kinase-MB (CK-MB) were measured on a Beckman Coulter AU680 autoanalyser (Beckman Coulter, Brea, CA, USA) at the Biochemistry Laboratory of Balıkesir University Health Practice and Research Hospital.

Trunk blood collection carries a recognised risk of in vitro haemolysis, which elevates LDH, AST and CK-MB without corresponding tissue injury. Samples were therefore screened against a single pre-specified quantitative criterion: an LDH activity exceeding five times the cohort median (749 IU/L; threshold 3745 IU/L). Four samples met this criterion—Control-6 (14,086 IU/L), HP + ILE-1 (16,129 IU/L), HP + ILE-4 (13,722 IU/L) and HP + ILE-7 (22,278 IU/L)—and these four also carried the four highest CK-MB values in the dataset, the pattern expected of haemolysis rather than of organ injury. The classification is insensitive to the choice of multiplier: any threshold between three and fifteen times the cohort median identifies exactly the same four samples, because the remaining 31 samples span 205–1200 IU/L and no sample lies between 1200 and 13,722 IU/L.

Because no conclusion should rest on a single handling decision, all haemolysis-sensitive analytes were analysed under four strategies: all samples retained; the four flagged samples excluded; 90th-percentile winsorisation; and rank transformation. The results are reported in Section 3.3 and and were concordant across all four strategies.

### 2.7. Oxidative Stress Assessment

Total antioxidant status (TAS) and total oxidant status (TOS) were determined using commercial automated colorimetric assays (Rel Assay Diagnostics, Gaziantep, Türkiye; catalogue numbers RL0017 and RL0024) according to the methods of Erel [26,27]. TAS was measured by the ABTS radical cation decolourisation method and expressed as mmol Trolox equivalent/L; TOS was measured by the ferrous ion–o-dianisidine oxidation method and expressed as µmol H_2_O_2_ equivalent/L. The oxidative stress index was calculated as follows:

OSI (arbitrary unit) = TOS (µmol H_2_O_2_ Eq/L)/[TAS (mmol Trolox Eq/L) × 10]

Spectrophotometric readings were performed on a Varioskan Flash multimode reader (Thermo Scientific, Waltham, MA, USA).

### 2.8. Histopathological Examination

Liver, lung, kidney, testis, heart, brain (left hemisphere) and both eyes were fixed in 10% neutral-buffered formalin, dehydrated through graded ethanol, cleared in xylene, embedded in paraffin and sectioned at 4 µm. Sections were stained with haematoxylin and eosin (H&E) and examined on an Olympus BX53 light microscope (Olympus Corporation, Tokyo, Japan) by a board-certified pathologist blinded to group allocation.

Seventeen parameters were scored semi-quantitatively as 0 = absent, 1 = mild, 2 = moderate, 3 = severe: hepatic inflammation, stromal expansion, congestion and degeneration; pulmonary inflammation, stromal expansion and congestion; renal inflammation, stromal expansion and congestion; cardiac congestion and degeneration; cerebral oedema and inflammation; and ocular degeneration and congestion. Testicular spermatogenesis was assessed using the modified Johnsen score (1–10, higher values indicating more complete spermatogenesis).

Collagen deposition cannot be reliably quantified on H&E-stained sections, and established fibrogenesis cannot occur within a 24 h exposure window. The parameter scored in liver and kidney is therefore designated stromal expansion rather than fibrosis. It denotes widening of the periportal (hepatic) or interstitial (renal) compartment by pale eosinophilic matrix, with separation of adjacent parenchymal elements, and makes no claim about collagen content. It was graded as follows:

0 (absent): periportal or interstitial compartment of normal width, with parenchymal elements closely apposed.

1 (mild): focal widening affecting fewer than one third of the portal tracts or interstitial fields examined, with slight separation of adjacent structures.

2 (moderate): widening affecting one third to two thirds of fields, with clear separation of parenchymal elements and readily visible eosinophilic matrix at 200×.

3 (severe): widening affecting more than two thirds of fields, with marked separation and distortion of the adjacent parenchymal architecture.

Whether this change represents oedema, early stellate or myofibroblast activation, or nascent matrix deposition cannot be resolved on H&E and would require Masson’s trichrome or picrosirius red staining with α-smooth muscle actin immunohistochemistry. Such confirmatory staining could not be performed, for the reason given in Section 2.11. The interpretation of this parameter is therefore bounded by what H&E permits, and is discussed further in Section 4.7.

### 2.9. Inter-Observer Reliability

To quantify the reproducibility of the ordinal scores, a stratified random sample of 12 animals (30% of the cohort; three per group) was independently re-scored by a second board-certified pathologist. Animals were selected using a fixed random seed to make the selection reproducible. Slides were relabelled with blind alphanumeric codes carrying no group information, and the second observer had access neither to the group allocation nor to the first observer’s scores. Both observers applied identical scoring definitions. All seven organs and all seventeen parameters were re-scored, giving 203 paired observations. Independent scores were retained unaltered for the reliability analysis; no consensus adjustment was applied.

### 2.10. Statistical Analysis

Analyses were performed in IBM SPSS Statistics v22.0 (IBM Corp., Armonk, NY, USA) with independent verification in Python 3.13 (SciPy 1.17). Normality was assessed by the Shapiro–Wilk test and homogeneity of variance by Levene’s test.

Ordinal histopathological scores are presented as median (minimum–maximum) and were compared across the four groups using the Kruskal–Wallis H test. Where the omnibus test was significant, pre-specified pairwise comparisons (control vs. HP, HP vs. HP + ILE, control vs. HP + ILE, control vs. ILE) were performed using the Mann–Whitney U test. Because seventeen parameters were tested, *p*-values within each family of comparisons were corrected using the Benjamini–Hochberg procedure controlling the false discovery rate at 5%; both uncorrected *p* and adjusted *q* values are reported. Effect sizes for ordinal comparisons are given as the rank-biserial correlation *r*.

Continuous biochemical variables are expressed as mean ± SD with 95% confidence intervals and were compared using one-way ANOVA where distributional assumptions were met, and by Kruskal–Wallis test otherwise; both are reported where they diverge. Pairwise comparisons used Welch’s *t*-test and the Mann–Whitney U test, with Cohen’s *d* as the effect size. Associations between histopathological scores and biochemical parameters were assessed by Spearman’s rank correlation, with the number of paired observations stated for every coefficient. Two-sided *p* < 0.05 was considered significant.

Inter-observer agreement was quantified as exact agreement, agreement within one grade, and weighted kappa. The weighting scheme was specified before analysis: linear weights for the 0–3 ordinal parameters and quadratic weights for the 1–10 modified Johnsen score. Two-sided 95% confidence intervals for kappa were obtained by bootstrap resampling (5000 replicates). Kappa is undefined where both observers assign a single invariant category; such parameters are reported as “not estimable” with the exact agreement still given.

Parameters with zero variance across all groups were not tested, since no rank statistic is defined; these are reported as “not evaluable”.

### 2.11. Availability of Archived Specimens

Histopathological scoring, including the independent re-scoring described in Section 2.9, was completed on the original stained sections at the time of the study. When those sections and the corresponding paraffin blocks were subsequently sought, in order to prepare additional photomicrographs and to attempt confirmatory special staining, they could not be located in the departmental archive.

The consequences are stated plainly. No further sections can be cut from this material; no special or immunohistochemical stains can be applied to it; no additional photomicrographs can be taken from any group; and the scoring cannot be repeated, extended or independently re-examined. The scores reported here, the inter-observer reliability analysis, and the five photomicrographs captured during the original assessment constitute the complete and final histopathological record of this study. The implications for interpretation are discussed in Section 4.7.

### 2.12. Use of Generative Artificial Intelligence

During the preparation of this manuscript, the authors used Claude (Anthropic, San Francisco, CA, USA USA) solely for English-language editing and stylistic refinement. The authors reviewed and edited all language suggestions and take full responsibility for the content of this publication.

## 3. Results

### 3.1. Clinical Observations

All forty animals survived to the 24 h endpoint. Haloperidol-treated animals (Groups 2 and 3) developed catalepsy, reduced locomotor activity and hypothermia within 1–2 h of dosing, consistent with the known pharmacodynamic profile of the drug; these signs had partially resolved by 8–12 h. No animal showed respiratory compromise or cardiovascular collapse. Animals receiving ILE alone were clinically indistinguishable from controls throughout.

### 3.2. Oxidative Stress Parameters

Oxidative stress data are presented in Table 2. Total oxidant status did not differ between groups (Kruskal–Wallis *p* = 0.431; ANOVA F (3,30) = 0.73, *p* = 0.542), and no pairwise comparison approached significance. The oxidative stress index showed a numerically graded pattern (control 0.154 < ILE 0.180 < HP 0.191 < HP + ILE 0.213) but no statistically detectable group effect (Kruskal–Wallis *p* = 0.512; ANOVA F (3,30) = 0.83, *p* = 0.486), and no pairwise contrast was significant (HP vs. control *p* = 0.315, *d* = 0.58; HP + ILE vs. control *p* = 0.193, *d* = 0.64).

Total antioxidant status did differ between groups (Kruskal–Wallis *p* = 0.028; Alexander–Govern *p* = 0.033). The contrast driving this effect was between the ILE-alone group and controls (1.13 ± 0.16 vs. 1.47 ± 0.18 mmol Trolox Eq/L; *p* = 0.011, *d* = −1.99); the HP + ILE group also showed lower TAS than controls, at the margin of significance (*p* = 0.070, *d* = −1.08), whereas haloperidol alone had no effect (*p* = 0.364).

With the available sample sizes (n = 7–10 per group), the study had approximately 80% power to detect a standardised difference of *d* ≈ 1.4 in OSI. The observed HP vs. control effect (*d* = 0.58) therefore cannot be excluded on the basis of these data; the correct interpretation is that no oxidative stress difference was detected, not that none exists.

### 3.3. Hepatic, Renal and Cardiac Biochemistry

Biochemical data are presented in Table 3.

Renal function. Serum urea showed a large and unambiguous group effect (ANOVA F (3,31) = 13.41, *p* < 0.001, η^2^_p_ = 0.565; Kruskal–Wallis *p* < 0.001). Urea was elevated in the HP group (67.4 ± 13.2 vs. 46.5 ± 8.8 mg/dL; Welch *p* = 0.001, Mann–Whitney *p* = 0.002, *d* = 1.82) and in the HP + ILE group (61.5 ± 8.7 mg/dL; *p* = 0.003, *d* = 1.72), and was unaffected by ILE alone (41.9 ± 3.5 mg/dL; *p* = 0.207). Critically, urea in the HP + ILE group did not differ from the HP group (*p* = 0.256), indicating that ILE did not correct the functional renal deficit. Creatinine did not differ between groups (ANOVA *p* = 0.176; Kruskal–Wallis *p* = 0.205), a dissociation consistent with tubulo-interstitial rather than glomerular injury.

Hepatic markers. Neither AST nor ALT was elevated by haloperidol. AST showed no group effect (ANOVA *p* = 0.439; Kruskal–Wallis *p* = 0.488). ALT likewise showed no omnibus effect (ANOVA *p* = 0.405; Kruskal–Wallis *p* = 0.220), and where a pairwise difference was detectable, it was in the opposite direction to that expected: ALT in the HP group was lower than in controls (43.5 ± 18.2 vs. 54.7 ± 14.8 IU/L; Mann–Whitney *p* = 0.021). ALP showed no significant omnibus effect (ANOVA *p* = 0.101; Kruskal–Wallis *p* = 0.097), with lower values in the HP + ILE group than controls on pairwise testing (*p* = 0.009). These findings are addressed in Section 4.3.

Cardiac markers and LDH. CK-MB did not differ between groups (ANOVA *p* = 0.069; Kruskal–Wallis *p* = 0.158), and LDH showed no group effect under any handling strategy.

The four samples flagged as haemolysed were analysed under four alternative strategies (Table 4). No analyte changed its significance status under any strategy: LDH, AST, CK-MB, ALT and ALP remained non-significant throughout, and urea remained highly significant throughout. Notably, LDH did not differ between groups even when all samples were retained (*p* = 0.109), so no conclusion in this manuscript depends on the decision to flag or exclude these four samples.

### 3.4. Histopathological Findings

**Figure 1:**
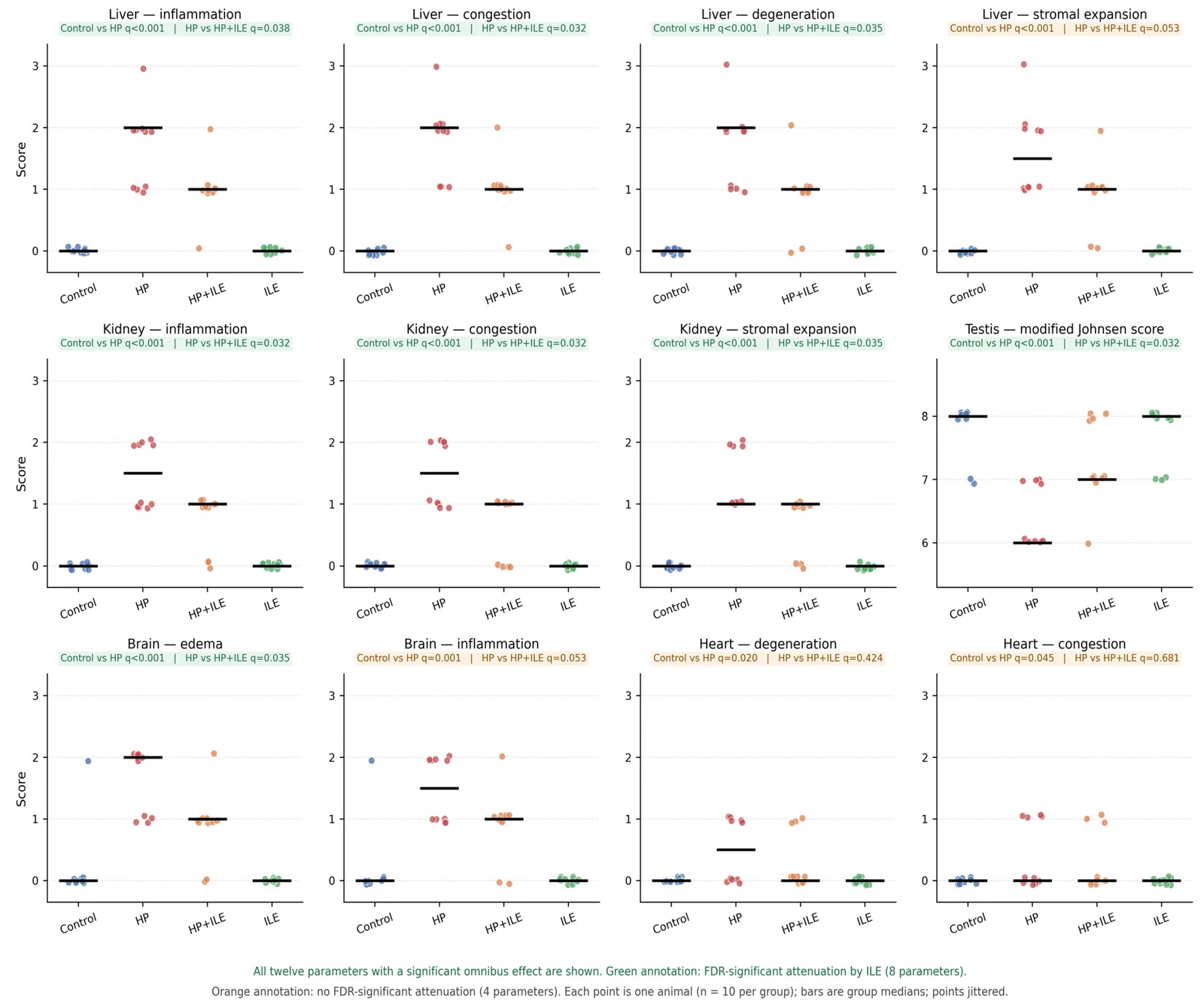
Individual animal scores for all twelve parameters with a significant omnibus effect, across all four experimental groups (n = 10 per group). Both treatment-responsive and non-responsive outcomes are shown: green annotations mark the eight parameters in which ILE co-administration produced FDR-significant attenuation, and orange annotations mark the four in which it did not (hepatic stromal expansion and cerebral inflammation, which showed the same direction of effect but did not survive FDR correction, and the two cardiac parameters, which were unaffected). Each point is one animal; horizontal bars are group medians. Points are jittered horizontally and vertically so that overlapping values remain visible. Annotations above each panel give the FDR-adjusted *q* value for control vs. HP and for HP vs. HP + ILE. The figure permits direct assessment of the magnitude of haloperidol-associated injury, the differential response following ILE co-administration, the residual difference from control in the HP + ILE group, and the absence of abnormality in the ILE-alone group. Underlying values for every animal and parameter are provided in Supplementary Table S1.

**Figure 2:**
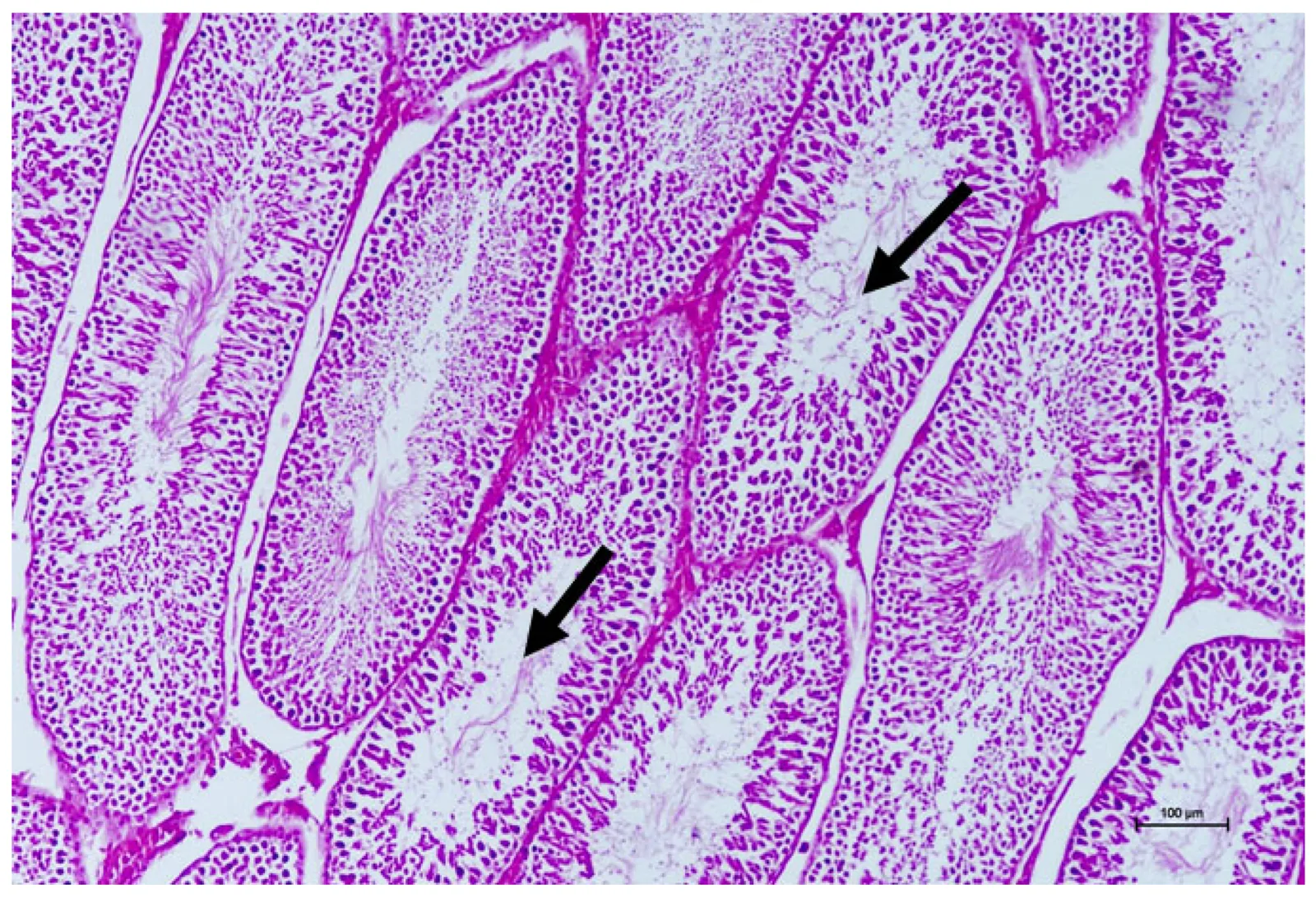
Testicular histopathology in the HP group showing reduced spermatogenesis (H&E, 200×). Arrows indicate seminiferous tubules with diminished germinal epithelium and reduced luminal spermatozoa compared with control tissue architecture. Scale bar = 100 µm.

**Figure 3:**
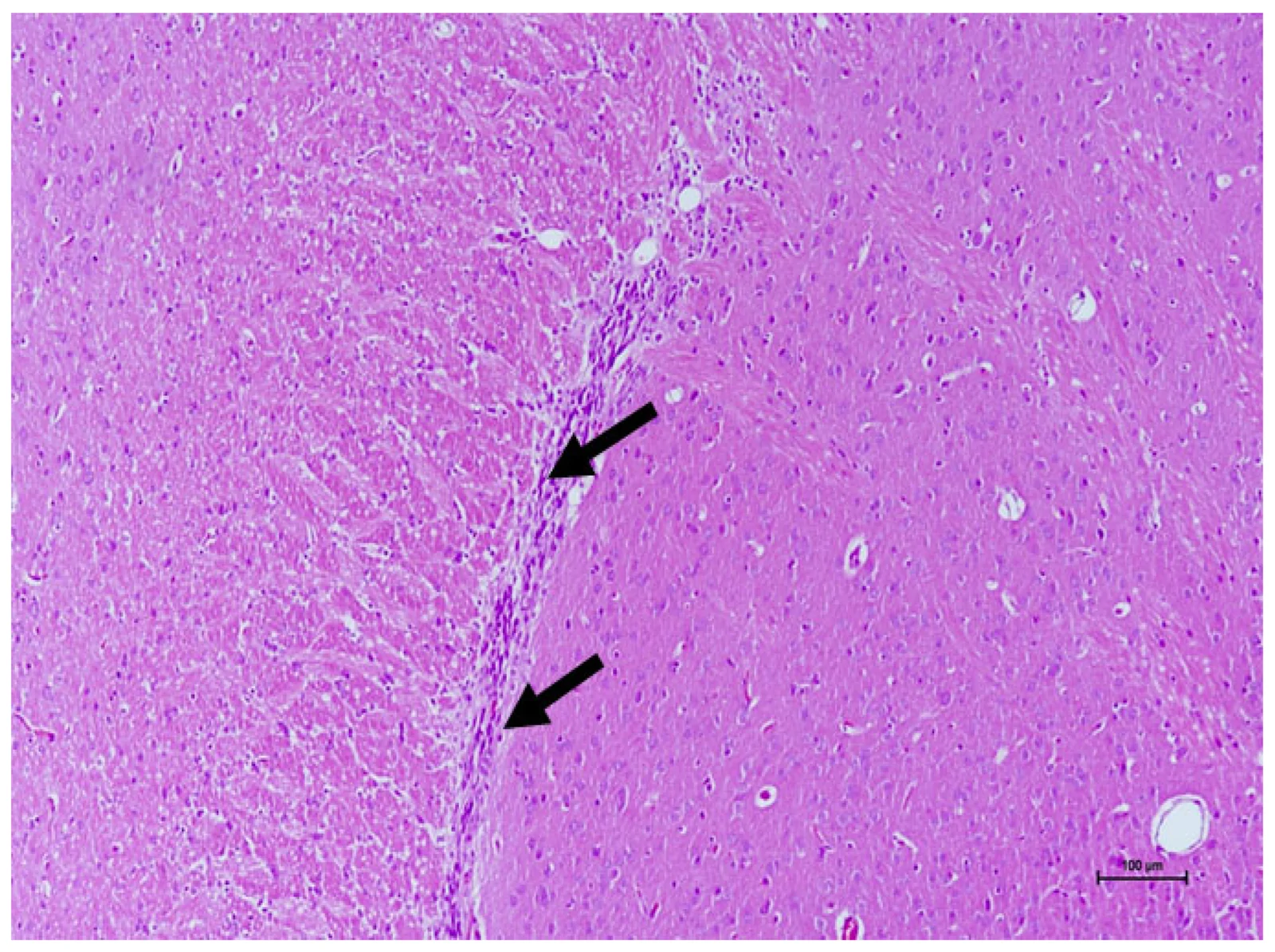
Cerebral tissue from the HP group showing inflammatory infiltration (H&E, 200×). Arrows indicate perivascular inflammatory cells and microglial activation. Scale bar = 100 µm.

**Figure 4:**
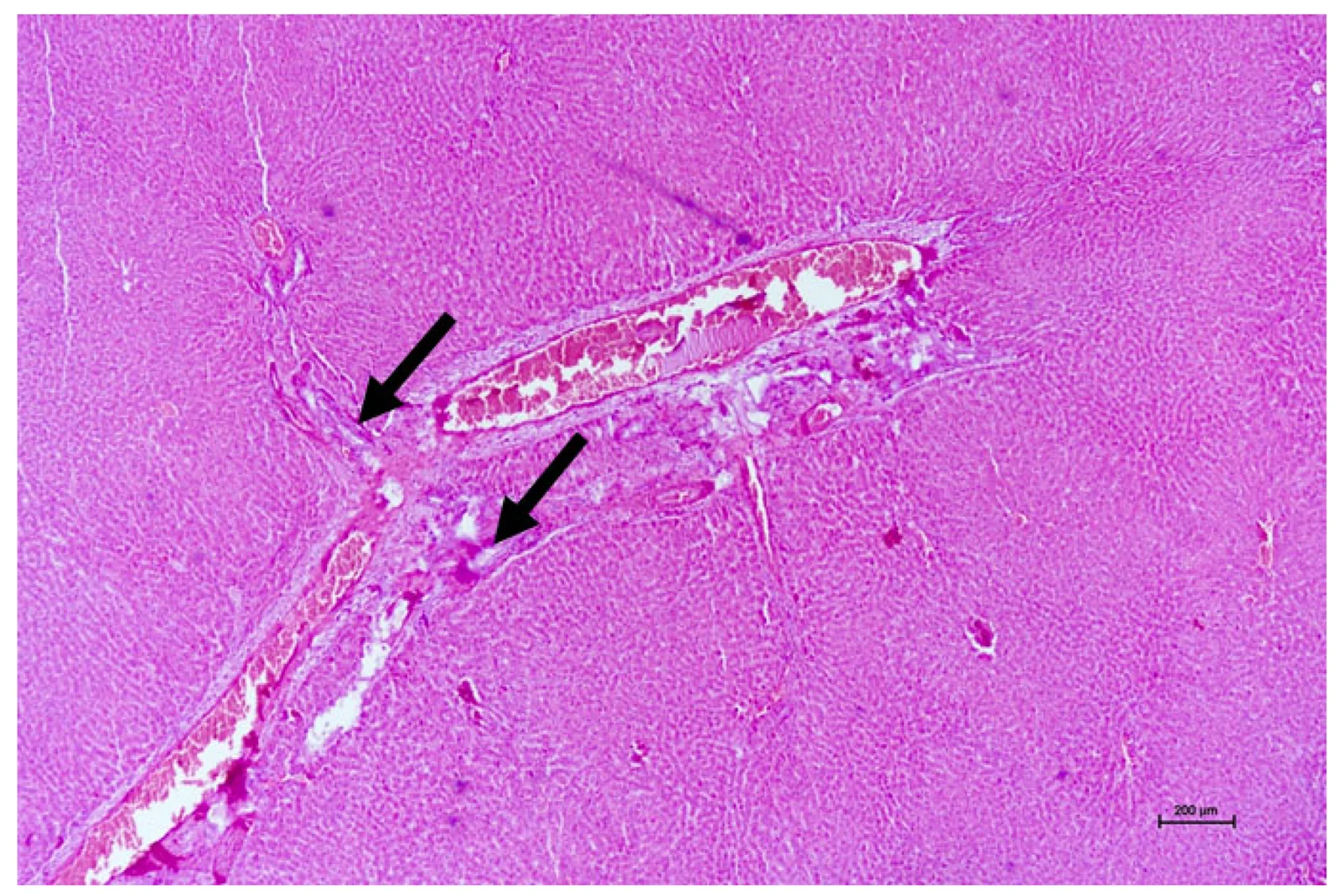
Hepatic periportal stromal expansion in the HP group (H&E, 200×). Arrows indicate increased periportal eosinophilic matrix with hepatocellular architectural disruption. Collagen content cannot be quantified on H&E sections (Section 4.7). Scale bar = 200 µm.

**Figure 5:**
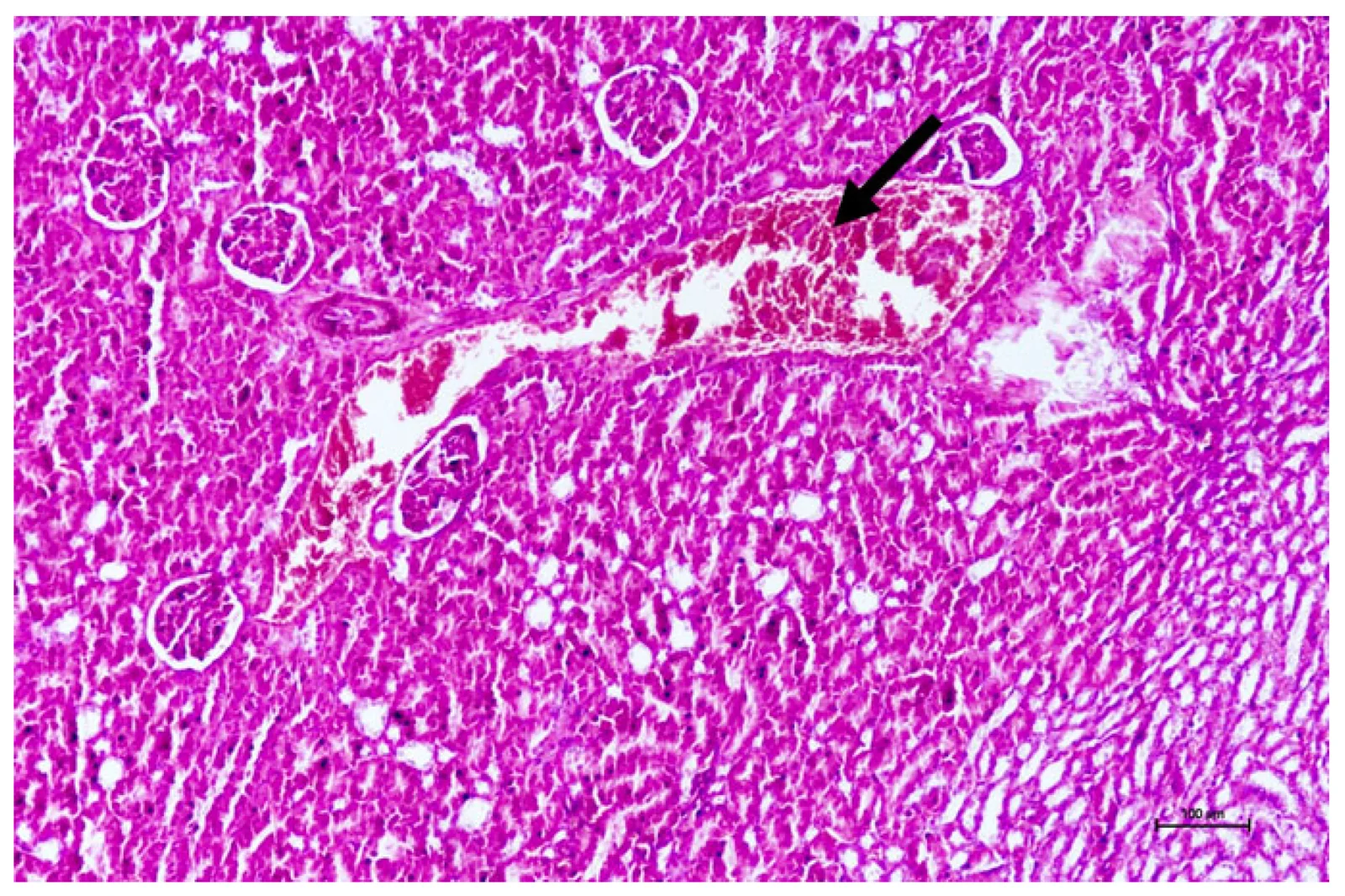
Renal congestion in the HP group (H&E, 200×). Arrows indicate vascular congestion in glomerular and peritubular capillaries with mild tubular dilatation.

**Figure 6:**
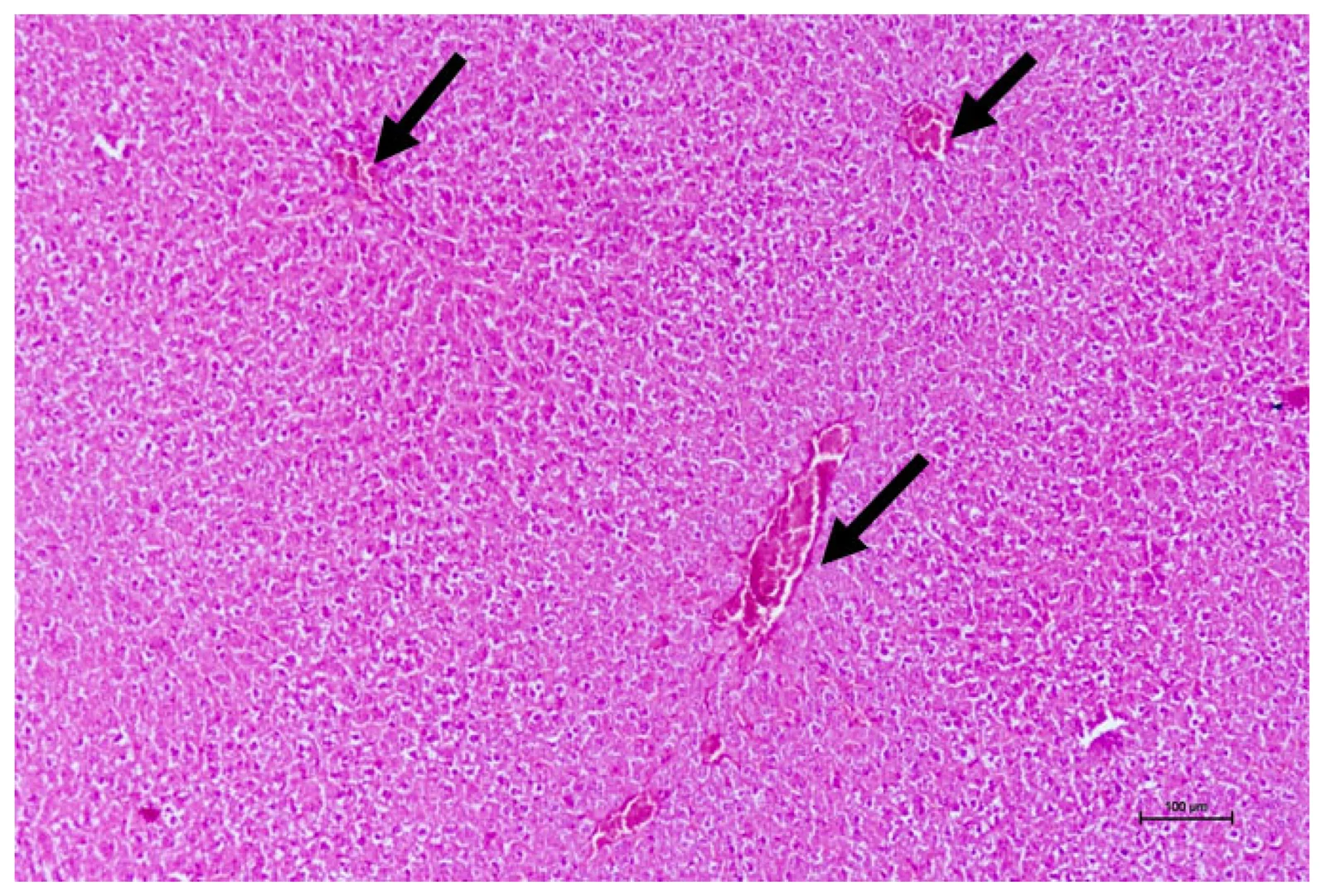
Hepatic congestion in the HP group (H&E, 200×). Arrows indicate central vein congestion with sinusoidal dilatation.

Complete histopathological results are presented in Table 5, and pairwise comparisons in Table 6. Because the archived sections could not be retrieved to prepare comparative photomicrographs across groups (Section 2.11), the distribution of individual animal scores is instead presented graphically in Figure 1, which permits direct visual assessment of the between-group differences at the level of individual animals. Representative photomicrographs of the haloperidol group, captured during the original assessment, are shown in Figure 2, Figure 3, Figure 4, Figure 5 and Figure 6.

Throughout this section, *stromal expansion* denotes acute widening of the periportal or interstitial compartment by pale eosinophilic matrix, as defined and graded in Section 2.8. It should not be read as established collagenous fibrosis, which cannot develop within 24 h of a single exposure and cannot be demonstrated on H&E.

Sixteen of the seventeen parameters were evaluable; ocular congestion scored zero in every animal in every group and was therefore not testable. After FDR correction, the Kruskal–Wallis test detected significant between-group differences for all four hepatic parameters, all three renal parameters, both cerebral parameters, both cardiac parameters and the testicular Johnsen score (all *q* ≤ 0.047). No pulmonary or ocular parameter differed between groups.

Haloperidol-induced injury (control vs. HP). Haloperidol produced severe multi-compartmental hepatic pathology, with median scores of 2 for inflammation, congestion and degeneration and 1.5 for stromal expansion (all *p* < 0.0001, *q* < 0.001). Renal inflammation and congestion (both median 1.5) and stromal expansion (median 1) were similarly elevated (all *p* < 0.0001, *q* < 0.001). Cerebral oedema (median 2) and inflammation (median 1.5) were significantly increased (*p* = 0.0005 and *p* = 0.0007). Spermatogenesis was impaired, with the modified Johnsen score falling from a control median of 8 to 6 (*p* = 0.0003). Cardiac changes were milder but statistically detectable: degeneration (median 0.5; *p* = 0.014, *q* = 0.020) and congestion (*p* = 0.034, *q* = 0.045). Pulmonary and ocular parameters were unaffected (all *q* > 0.19).

One control animal showed moderate cerebral oedema and inflammation (score 2 for both), accounting for the control range of 0–2 for these parameters; no other control animal showed any abnormality in any organ.

Effect of ILE co-administration (HP vs. HP + ILE). ILE significantly attenuated haloperidol-associated injury in eight of the twelve affected parameters after FDR correction, with medium-to-large effect sizes (Table 6). The largest effects were on renal congestion (*p* = 0.004, *q* = 0.032, *r* = 0.70), the testicular Johnsen score (*p* = 0.008, *q* = 0.032, *r* = 0.66), renal inflammation (*p* = 0.006, *q* = 0.032, *r* = 0.65) and hepatic congestion (*p* = 0.007, *q* = 0.032, *r* = 0.64). Hepatic degeneration (*q* = 0.035), renal stromal expansion (*q* = 0.035), cerebral oedema (*q* = 0.035) and hepatic inflammation (*q* = 0.038) were also significantly reduced. Hepatic stromal expansion (*p* = 0.031, *q* = 0.053) and cerebral inflammation (*p* = 0.033, *q* = 0.053) showed the same direction of effect but did not survive FDR correction. Neither cardiac parameter was modified by ILE (*p* = 0.398 and *p* = 0.681).

Residual difference from control (control vs. HP + ILE). The improvement was incomplete in most organs. Hepatic (*p* ≤ 0.0005), renal (*p* ≤ 0.005) and cerebral (*p* = 0.008) parameters in the HP + ILE group remained significantly above control values, indicating residual injury. The testis was the only affected parameter for which the difference from control did not reach significance (median 7 vs. 8, *p* = 0.072). This is a significant improvement relative to the HP group (*q* = 0.032) but is not evidence of normalisation or of equivalence to control: a non-significant contrast at *p* = 0.072 with n = 10 per group is compatible with a residual deficit, and no equivalence testing was performed. Cardiac parameters in the HP + ILE group also did not differ from control (*p* = 0.077), reflecting the small magnitude of the initial cardiac insult rather than a treatment effect.

Safety of ILE alone (control vs. ILE). No animal receiving ILE alone showed any abnormality in any organ. Scores were identical to control for every parameter except the Johnsen score, which did not differ from control (*p* = 0.651), and the two cerebral parameters, where the ILE group scored uniformly zero and the control group contained the single outlying animal described above (*p* = 0.368). ILE alone therefore produced no detectable histopathological toxicity at the dose and schedule used.

### 3.5. Inter-Observer Reliability of Histopathological Scoring

Twelve animals (three per group, 30% of the cohort) were independently re-scored by a second blinded pathologist, yielding 203 paired observations across all seventeen parameters. Exact agreement was 95.6% (194/203), agreement within one grade was 100%, and no paired observation differed by two or more grades.

Weighted kappa was estimable for 13 of the 17 parameters (Table 7). The median weighted kappa was 0.893 (range 0.625–1.000). Eleven of the thirteen estimable coefficients fell in the “almost perfect” range (κ ≥ 0.81), and all thirteen were substantial or better (κ ≥ 0.61). Agreement was perfect (κ = 1.000) for renal stromal expansion, cardiac degeneration, cerebral oedema, cerebral inflammation and the testicular Johnsen score. The lowest coefficient, for pulmonary inflammation (κ = 0.625), reflects a single one-grade disagreement in a parameter where 11 of 12 animals scored zero; kappa is unstable under such skewed marginal distributions, and the exact agreement for this parameter was 91.7%.

Kappa was not estimable for pulmonary stromal expansion, pulmonary congestion, ocular degeneration and ocular congestion, in each case because both observers assigned the same single category to all twelve animals. Exact agreement for these four parameters was 100%.

Confidence intervals are wide, as expected with twelve animals, and should be read as indicating that the point estimates are compatible with substantial-to-perfect agreement rather than as precise estimates. The intervals for the parameters carrying the study’s principal conclusions—hepatic, renal, cerebral and testicular scores—exclude values below 0.49.

### 3.6. Structure–Function Relationships

Spearman correlations were computed between histopathological scores and biochemical values in animals for which both were available (Table 8).

Renal histopathology correlated strongly and consistently with serum urea: renal inflammation ρ = 0.735, renal congestion ρ = 0.689 and renal stromal expansion ρ = 0.597 (all *p* ≤ 0.0002, n = 35). This provides independent structural corroboration of the functional renal finding.

In contrast, hepatic histopathology showed no relationship with transaminase activity. Correlations between hepatic stromal expansion and ALT (ρ = −0.022, *p* = 0.902), hepatic inflammation and ALT (ρ = −0.159, *p* = 0.362), hepatic degeneration and AST (ρ = −0.097, *p* = 0.578), and hepatic congestion and AST (ρ = −0.165, *p* = 0.344) were all negligible and non-significant. A weak inverse association was observed between hepatic inflammation and ALP (ρ = −0.342, *p* = 0.044), which should be interpreted cautiously given the number of correlations examined.

Similarly, no association was found between the oxidative stress index and cerebral inflammation (ρ = 0.008, *p* = 0.963), cerebral oedema (ρ = 0.018, *p* = 0.920) or the testicular Johnsen score (ρ = −0.040, *p* = 0.821), n = 34. The Johnsen score correlated inversely with serum urea (ρ = −0.451, *p* = 0.007, n = 35), which most plausibly reflects shared dependence on haloperidol exposure rather than a mechanistic link between renal and testicular injury.

### 3.7. Representative Histopathology

The five photomicrographs below were captured from haloperidol-group sections during the original histopathological assessment. Because the archived material could not subsequently be retrieved (Section 2.11), no matched images from the control, HP + ILE, or ILE groups are available, and these figures should be read as illustrations of the lesions scored in the HP group rather than as a visual comparison between groups. The between-group comparison is presented quantitatively in Figure 1 and Table 5 and Table 6, and at the level of individual animals in Supplementary Table S1.

## 4. Discussion

This study is an acute rodent overdose model. A single oral dose of 17.5 mg/kg was chosen to produce an overt toxic syndrome; it is roughly fifty times the field doses used to tranquillise roe deer and bears no relation to any dose a veterinarian would administer. The endpoint is 24 h, the design is single-dose, and no target veterinary species was studied. Within those boundaries, it provides the first simultaneous, organ-by-organ characterisation of butyrophenone overdose and of the effect of intravenous lipid emulsion across seven organ systems in a single experiment, with histopathological, biochemical and oxidative endpoints measured in the same animals. Its function is to map which organ systems respond to lipid rescue and which do not, and thereby to generate testable questions for veterinary evaluation—not to supply evidence for the clinical use of ILE in animal patients. Because haloperidol is itself administered to animals in wildlife practice, because antipsychotics are a recognised cause of accidental toxicosis in companion animals, and because lipid rescue is used routinely in small-animal emergency medicine on an evidence base composed almost entirely of clinical case reports, these results speak to veterinary as well as human toxicology; the veterinary implications are set out in Section 4.6.

Four findings emerge. First, a single high oral dose of haloperidol produces reproducible microscopic injury within 24 h in the liver, kidney, brain and testis, together with a large elevation in serum urea, and milder cardiac involvement; the lung and eye are unaffected. Second, ILE co-administration significantly attenuates microscopic injury in eight of the twelve affected parameters across four organs, while four parameters—hepatic stromal expansion, cerebral inflammation and both cardiac endpoints—showed no FDR-significant attenuation; this differential pattern is described at 24 h, and its mechanism is not established here, with the largest single improvement in the testicular endpoint. Third, this histopathological protection is not accompanied by functional renal improvement: serum urea in the HP + ILE group remained as elevated as in the HP group despite significantly better renal histology. Fourth, neither systemic oxidative stress indices nor hepatic transaminases tracked the organ injury observed in the same animals.

### 4.1. Pattern of Haloperidol-Induced Multi-Organ Injury

The hepatic pattern documented here—inflammation, congestion, degeneration and periportal stromal change at median grades of 1.5–2—is consistent with haloperidol’s recognised hepatotoxic potential. The liver is the principal site of haloperidol metabolism, and reactive metabolite formation provides a plausible route to oxidative hepatocellular injury. Halici et al. [22] and Hanagama et al. [28] reported comparable hepatic changes after chronic haloperidol administration; the present data indicate that qualitatively similar lesions are already established 24 h after a single high dose.

Renal involvement was equally consistent, affecting all three scored parameters, and was corroborated functionally by a large rise in serum urea (η^2^_p_ = 0.565) and structurally by strong correlations between renal scores and urea concentration (ρ = 0.60–0.74). The preservation of creatinine despite elevated urea points to tubulo-interstitial rather than glomerular injury, and the prominence of congestion among the renal findings is compatible with a haemodynamic contribution through renal vasoconstriction. This is the most robust and internally coherent finding of the study, supported by convergent structural and functional evidence.

Impairment of spermatogenesis, with the modified Johnsen score falling by two grades, is consistent with the established effects of D2-receptor blockade on the hypothalamic–pituitary–gonadal axis mediated by hyperprolactinaemia, and with reactive-oxygen-species-mediated damage to gonadal supporting cells reported for haloperidol and clozapine in ovarian tissue [23], and with the impairment of sperm parameters, testosterone and testicular antioxidant defences demonstrated for haloperidol in male rats [24]. Cerebral oedema and neuroinflammation reflect haloperidol’s ready passage across the blood–brain barrier. The absence of pulmonary and ocular findings, despite systematic scoring, is itself informative: the pattern of injury differs between organs, in a distribution broadly consistent with tissue perfusion and drug distribution rather than with a non-specific systemic response.

### 4.2. Differential Histopathological Response Among Organs

The central finding of this study is that the histopathological response to ILE co-administration extends well beyond the liver, and that it differs between organs at the 24 h endpoint. We describe this deliberately as a differential histopathological response rather than as organ-selective protection. The latter is a mechanistic claim, and it is not one these data can support: a single 24 h endpoint cannot distinguish genuine differences in protection from differences in lesion onset, recovery kinetics, tissue sensitivity, or the sensitivity of the individual scoring endpoints themselves. Because the ILE schedule was empirical and neither pharmacokinetic nor tissue drug measurements were obtained (Section 2.3 and Section 4.7), the mechanism underlying the differential pattern remains undetermined. Establishing organ selectivity as a mechanism would require serial endpoints and tissue concentration data. Significant attenuation was observed in the kidney (all three parameters), liver (three of four after FDR correction), brain (edema) and testis, with rank-biserial effect sizes of 0.55–0.70. The largest single effect was renal (*r* = 0.70 for congestion).

This broader pattern partially reconciles the previously discordant literature. Ghaleb et al. [17] reported hepatic and renal improvement after 10 mL/kg ILE in haloperidol-poisoned rats, which the present data confirm and extend to cerebral and testicular endpoints. Moshiri et al. [18] found that the optimal ILE dose differed between neurological and cardiac endpoints in rabbits; the present finding of a cerebral response alongside the complete absence of a cardiac response at a single dose is consistent with that endpoint-dependence, although it does not by itself establish its cause.

Under a strict “lipid sink” model, protection should scale with the degree to which lipid-phase partitioning reduces tissue drug exposure, which depends on organ blood flow, capillary architecture and diffusion barriers [15,29]. The organs responding here differ widely in these properties, which makes a purely sink-based explanation incomplete—but, for the reasons given above, the observed pattern is compatible with several non-mechanistic explanations and should not be read as evidence for or against the lipid sink model. The magnitude of the improvement in the testicular Johnsen score is notable, since the testis is rarely examined in lipid-rescue studies. Two cautions attach to it. A non-significant difference from control (*p* = 0.072) is not evidence of equivalence, so the improvement should not be described as normalisation. And, as set out in Section 4.7, the animals were 7–8 weeks old with control Johnsen scores already below fully mature values, so preservation or continuation of ongoing spermatogenic maturation cannot be distinguished from recovery of established testicular injury in this design. The observation warrants independent confirmation in sexually mature animals.

### 4.3. Structure–Function Dissociations

Two dissociations in these data have implications beyond the specific question of lipid rescue.

Renal histology improved without functional improvement. ILE significantly reduced renal inflammation, congestion and stromal expansion (*q* = 0.032–0.035), yet serum urea in the HP + ILE group was statistically indistinguishable from the HP group (*p* = 0.256) and remained significantly above control (*p* = 0.003). Histological improvement therefore did not translate into functional recovery within the 24 h observation window. Several explanations are possible: functional recovery may lag structural repair; urea elevation may be driven partly by prerenal factors (reduced intake, catabolic state) not addressed by ILE; or the semi-quantitative score may be insufficiently sensitive to the tubular changes that determine urea handling. Whichever applies, the observation is a caution against inferring functional benefit from histological improvement—a common inference in the lipid-rescue literature.

Transaminases were blind to established hepatic injury. Neither AST nor ALT was elevated in the haloperidol group; ALT was, in fact, significantly *lower* than in controls (*p* = 0.021), and correlations between hepatic scores and transaminase activity were essentially zero (|ρ| ≤ 0.17, all *p* > 0.34). This is not a null result of low interest. It indicates that in this model the serum enzymes conventionally used as hepatic injury markers fail to detect unequivocal microscopic hepatic pathology. Transaminase release requires hepatocyte membrane rupture, whereas the dominant lesions here were inflammatory, congestive and degenerative rather than frankly necrotic; the 24 h sampling point may also precede peak enzyme release, and the marked congestion may have impaired enzyme washout into the systemic circulation.

This interpretation is strictly bound to the 24 h sampling point, and the temporal argument deserves to be made explicitly. In rodent models of chemically induced hepatocellular injury, serum transaminase activity typically rises within 6–24 h and peaks between 24 and 48 h, with the exact profile depending on the toxicant, the dose and the dominant mode of cell death; predominantly necrotising injuries peak earlier than inflammatory or degenerative ones. Haloperidol undergoes extensive hepatic metabolism, and a single oral dose produces sustained rather than instantaneous hepatic exposure, so the injurious process was probably still evolving when the animals were sampled. A 48 or 72 h endpoint might therefore have captured a delayed enzyme peak corresponding to the structural damage already visible at 24 h, and the present design cannot exclude that possibility. What can be said is narrower and still useful: at 24 h, the point at which a poisoned patient is most likely to be sampled, transaminase activity did not reflect concurrent and unequivocal hepatic pathology.

The practical implication is directly relevant to clinical and forensic toxicology: normal transaminases 24 h after haloperidol overdose do not exclude significant hepatic injury, and serial rather than single measurement would be required to exclude it.

### 4.4. Absence of a Detectable Oxidative Stress Signal

Much of the mechanistic literature on haloperidol toxicity is framed around oxidative stress [19,20,21]. In the present study, no group difference was detected in total oxidant status (*p* = 0.431) or in the oxidative stress index (*p* = 0.512), and the OSI showed no correlation with cerebral or testicular injury scores in the same animals (|ρ| ≤ 0.04).

This null result cannot be attributed to anaesthetic confounding, since terminal anaesthesia was deliberately avoided precisely to protect the validity of these measurements (Section 2.4)—a design choice that removes one of the commonest sources of artefact in rodent oxidative stress studies and makes the negative finding more, rather than less, interpretable.

Two interpretations must therefore be distinguished. The first is that serum oxidative indices are simply insensitive to compartmentalised tissue-level oxidative injury: TOS and TAS integrate whole-body redox status and may be dominated by circulating antioxidants unrelated to events in target organs. The second is that oxidative stress is not a major mediator of the injury observed here. These data cannot discriminate between them, and the study was underpowered for the observed effect sizes (approximately 80% power for *d* ≈ 1.4, against an observed HP vs. control OSI effect of *d* = 0.58). What can be stated is bounded by what was measured. Only serum TOS, TAS and OSI were assayed at a single time point in 34 animals. No tissue oxidative marker was determined—no malondialdehyde, reduced glutathione, superoxide dismutase or catalase in liver, kidney, brain or testis, and no inflammatory cytokine or apoptotic marker. These data therefore do not exclude tissue-level oxidative injury, and should not be read as evidence that oxidative stress is not involved in haloperidol toxicity. The claim supported by this study is the narrower one: serum TOS, TAS and OSI did not reflect concurrent organ-level injury at 24 h and did not function as usable surrogate endpoints in this model. Studies relying on serum oxidative indices alone to infer organ-level oxidative injury should be interpreted with corresponding caution, and tissue-level markers should be measured in future work.

The one significant oxidative finding was a reduction in total antioxidant status in the ILE-alone group relative to controls (*p* = 0.011, *d* = −1.99), with the HP + ILE group intermediate (*p* = 0.070). Three explanations must be distinguished, and these data cannot separate them. The first is analytical rather than biological. TAS was measured by ABTS radical cation decolourisation, a spectrophotometric method that reads an absorbance change in serum; lipaemic serum scatters light and raises background absorbance, and lipaemia is an expected consequence of two 7.5 mL/kg infusions of a 20% emulsion, the second given 16 h before sampling. Spectrophotometric interference from residual lipaemia could therefore have depressed the apparent TAS in exactly the two groups that received lipid, which is precisely the pattern observed (ILE lowest, HP + ILE intermediate, the lipid-free groups unaffected). Serum was not graded for lipaemia at the time of assay and triglyceride concentrations were not measured, so this cannot be excluded and is arguably the most parsimonious explanation. The second is dilutional: the infused volume may have diluted circulating antioxidants. The third is genuine consumption of antioxidant capacity through peroxidation of infused polyunsaturated fatty acids, lipid emulsion not being itself an antioxidant. Because this finding rests on seven animals per group and was not accompanied by any change in TOS or OSI, and because the ILE-alone group showed no histopathological abnormality whatsoever, it should be regarded as hypothesis-generating and requires replication before any inference about ILE-related oxidative burden is drawn.

### 4.5. Absence of Cardiac Benefit and Clinical Implications

Cardiac degeneration and congestion were significantly increased by haloperidol but entirely unaffected by ILE (*p* = 0.398 and *p* = 0.681), and CK-MB did not differ between groups. This is the finding with the most direct bearing on clinical practice, because the principal rationale for ILE in lipophilic drug poisoning is cardiovascular rescue.

The mildness of the cardiac insult itself warrants comment, given haloperidol’s reputation for cardiotoxicity. That reputation rests on electrophysiological rather than structural toxicity: QTc prolongation and torsades de pointes arise from blockade of the rapid delayed-rectifier potassium current, a functional effect on repolarisation with no histological correlate. A drug can therefore be profoundly cardiotoxic in the clinically relevant sense while leaving myocardium that appears near-normal under the microscope. Two further factors are likely to have contributed. The rat has a ventricular repolarisation profile markedly different from that of humans and dogs, with minimal contribution from the rapid delayed-rectifier current, and is for that reason a poor model of drug-induced QT prolongation. And 24 h after a single dose is a short window for structural myocardial lesions, which typically require sustained ischaemic or metabolic stress. The mild congestion and degeneration observed are therefore best read as non-specific consequences of systemic haemodynamic disturbance rather than as a structural signature of haloperidol cardiotoxicity.

Two caveats temper the conclusion about ILE. First, the cardiac insult in this model was mild (median degeneration score 0.5), leaving limited room to demonstrate improvement. Second, the endpoint was structural rather than functional: no electrocardiographic or haemodynamic measurements were made, and haloperidol’s clinically important cardiac toxicity is electrophysiological (QTc prolongation, torsades de pointes) rather than histological. A model incorporating continuous ECG monitoring would be required to test cardiac benefit properly.

These caveats bound the claim tightly. What these data establish is that ILE did not modify the mild histological cardiac changes observed in this model; whether ILE confers functional or electrophysiological cardiac protection in haloperidol toxicity remains untested and cannot be inferred either way from this study. Because the clinically important cardiotoxicity of haloperidol is electrophysiological, the question that matters most for practice is precisely the one this design could not address. Read narrowly, these data provide no positive support for extending ILE indications to routine use in haloperidol overdose on cardiac grounds, but neither do they exclude such a benefit. They are consistent with the position statement of the American College of Medical Toxicology [30], which restricts ILE to local anaesthetic systemic toxicity and to lipophilic cardiotoxin exposures unresponsive to conventional therapy. Any consideration of off-label use must weigh the recognised complications of high-dose lipid infusion, including hypertriglyceridaemia, pancreatitis, acute lung injury, fat overload syndrome and interference with laboratory assays [31,32]. Notably, the present study found no histopathological toxicity attributable to ILE alone, which is reassuring as far as it goes but does not address these systemic complications, none of which were assessed.

The reliability analysis supports the interpretation of these ordinal endpoints. Independent re-scoring of 30% of the cohort by a second blinded pathologist produced 95.6% exact agreement with no disagreement exceeding one grade, and weighted kappa coefficients that were substantial or better for every estimable parameter (median κ = 0.893). Agreement was perfect for cerebral oedema, cerebral inflammation, cardiac degeneration, renal stromal expansion and the testicular Johnsen score. Semi-quantitative scoring is often criticised as subjective; in this dataset the scoring was highly reproducible, and the between-group differences reported above are therefore unlikely to reflect observer variation.

### 4.6. Implications for Veterinary Toxicology and Emergency Practice

Four aspects of these results bear directly on veterinary practice.

Organ-level evidence for a drug class used in animals. Haloperidol is administered to wild ungulates during capture and translocation, and azaperone to swine, yet the consequences of supratherapeutic butyrophenone exposure at tissue level have not previously been described. The present data show that a single high dose produces reproducible injury in liver, kidney, brain and testis within 24 h, with cerebral oedema and neuroinflammation among the most severe findings. This is relevant to a setting in which neuroleptised animals are simultaneously exposed to transport stress, hyperthermia and dehydration—precisely the conditions under which the extrapyramidal and paradoxical reactions reported in treated wild species have been observed [1,3]. The dose used here (17.5 mg/kg) greatly exceeds the field doses reported for roe deer (approximately 0.3 mg/kg) [2], so these findings describe overdose rather than routine tranquillisation; they nonetheless define the organ systems that merit attention when a butyrophenone exposure is suspected to have been excessive.

Controlled tissue-level data on ILE, which the veterinary literature currently lacks. Lipid rescue is used routinely in small-animal emergency medicine, but its evidence base is dominated by case reports with clinical endpoints [8,9]. To our knowledge this is the first controlled study to ask, across multiple organs simultaneously and with blinded scoring, what ILE does to the tissues. The answer—attenuation in liver, kidney, brain and testis, no detectable effect on the two cardiac measures, and incomplete restoration in most organs—is a description of the tissue-level response in this model. It is not an endorsement of lipid rescue in veterinary patients, and we do not offer one: an acute high-dose rodent model assessed at a single endpoint cannot establish therapeutic benefit in any species. What it provides is a first tissue-level description against which clinical case material can be interpreted. The differential pattern observed here suggests one hypothesis for the variable outcomes reported in veterinary case series—that whether ILE appears to work may depend on which organ system dominates the clinical picture—but this remains a hypothesis requiring temporal and pharmacokinetic evidence to test.

Absence of histopathological injury from ILE alone, and what it does not establish. Veterinary ILE is frequently given empirically to a deteriorating patient, sometimes before the toxicant is identified, so any controlled safety observation is useful. The ILE-alone group here showed no histopathological abnormality in any of the seven organs examined, with scores identical to control across every parameter.

The limits of that observation must be stated plainly. It applies to one dose (7.5 mL/kg × 2), one formulation (Clinoleic, 80% olive oil/20% soybean oil), one species, one sex, one age and one time point (24 h). It does not establish the general safety of ILE in veterinary use, because the adverse effects that actually matter clinically were not assessed: hypertriglyceridaemia, gross lipaemia, pancreatitis, fat overload syndrome, pulmonary complications, interference with laboratory assays, and any delayed toxicity arising beyond 24 h [10,31,33]. Several of these produce no histological lesion in the organs examined and would be invisible to this study’s endpoints by design. Indeed, the reduction in measured TAS in this same group (Section 4.4) may itself reflect assay interference from residual lipaemia—an example of exactly the kind of effect that this design detects only obliquely. The correct summary is that ILE alone caused no detectable histopathological injury under the specific dose, formulation, species and 24 h conditions used here, which is a necessary but not sufficient component of a safety assessment.

Hypotheses concerning routine clinical monitoring. Three observations generate hypotheses for veterinary investigation. They are stated as hypotheses rather than as practice guidance, because a rodent overdose model cannot establish how a marker behaves in a poisoned dog, cat or ungulate. First, hepatic transaminases were not elevated despite unequivocal hepatic pathology and showed no correlation with it; whether normal ALT and AST at 24 h can likewise fail to exclude hepatic injury in a target species is a question these data raise but cannot answer. Second, improved renal histology was not accompanied by improved serum urea, which suggests, without establishing, that structural and functional renal recovery may dissociate after lipid rescue; confirmation would require serial functional measurement in a target species. Third, serum oxidative stress indices did not track organ injury in this model, and their analytical behaviour in lipaemic samples (Section 4.4) warrants examination before they are relied upon in veterinary toxicological studies. None of these observations should alter clinical monitoring practice on the strength of the present data.

Extrapolation across species requires care. The rat differs from the dog, cat and wild ungulate in hepatic metabolic capacity, dopaminergic receptor distribution, lipid clearance and susceptibility to capture myopathy, and the ILE regimen used here (7.5 mL/kg × 2) is not the protocol used in small-animal practice. These results therefore identify which organs respond and which do not, and generate hypotheses for veterinary evaluation; they do not establish a dosing recommendation for any veterinary species.

### 4.7. Limitations

First, the single-dose 24 h design models acute overdose and does not address chronic antipsychotic toxicity or the subacute presentations more commonly encountered clinically. The observation window may also have been too short to capture peak biochemical change, as discussed in Section 4.3.

Second, and pervading several of the limitations that follow, the archived sections and paraffin blocks from this study could not be located when they were sought after the primary analysis was complete (Section 2.11). Three specific consequences follow. Confirmatory special staining of the stromal expansion parameter is not possible on this material. Comparative photomicrographs across groups cannot be prepared, so between-group differences can be presented numerically and graphically but not shown histologically. And the histopathological scores cannot be independently re-examined by a third party, which places more weight than would be ideal on the inter-observer reliability sub-study performed while the material was still available. Readers should weigh the histopathological findings accordingly. The individual animal scores are provided in full as Supplementary Table S1, so that every reported statistic can at least be recomputed from the underlying data.

Third, the parameter reported as stromal expansion cannot be equated with fibrosis. Collagen deposition is not quantifiable on H&E-stained sections, and established fibrogenesis cannot occur within 24 h of a single exposure. The scored changes most plausibly represent periportal or interstitial stromal expansion, oedema and early stellate or myofibroblast activation. Confirmation would require Masson’s trichrome or picrosirius red staining, ideally with α-smooth muscle actin immunohistochemistry.

Fourth, control animals showed modified Johnsen scores of 7–8 rather than the 9–10 expected in fully mature rats. The animals were 7–8 weeks old, an age at which spermatogenesis in the rat is still being established, so baseline spermatogenic maturity was submaximal. This has two consequences that go beyond a compressed dynamic range.

First, a rapidly expanding germinal epithelium and a mature one are not equivalent targets. The pubertal testis has a high proportion of mitotically active spermatogonia and differentiating spermatocytes, populations more vulnerable to an acute insult than the quiescent stem-cell compartment of a mature testis, while also retaining greater capacity for rapid repopulation. Hyperprolactinaemia from D2-receptor blockade acts on a hypothalamic–pituitary–gonadal axis that is itself still maturing, and the consequences of transient axis suppression during maturation may differ in kind, not merely in degree, from suppression of an established axis. Lipid emulsion may also interact differently with a developing testis: testicular perfusion, blood–testis barrier integrity and lipid handling all change across puberty.

Second, and directly relevant to interpretation, the improvement in the Johnsen score in the HP + ILE group cannot be distinguished on these data from preservation or continuation of an interrupted maturational trajectory. If haloperidol transiently arrested ongoing maturation and ILE reduced the effective testicular drug exposure sufficiently for maturation to continue, the same 24 h endpoint would result. Structural protection of an established epithelium and preservation of an ongoing developmental process would look identical in a single cross-sectional score. Distinguishing them requires replication in animals older than 10 weeks, ideally with staged tubule analysis, hormonal measurements and a later endpoint capable of showing whether the recovery persists.

Fifth, biochemical and oxidative endpoints were available for 35 and 34 of the 40 animals respectively, and sample loss was unequally distributed between groups. The direction of that inequality is worth stating, because it bears on the most obvious bias hypothesis. Sample loss occurred exclusively in the control and ILE groups; every animal in the HP and HP + ILE groups yielded complete data (Fisher’s exact test, *p* = 0.047 for biochemistry, *p* = 0.020 for oxidative stress; Table 1). Attrition was therefore not associated with haloperidol exposure, and cannot be explained by drug-induced hypothermia, systemic congestion or haemodynamic compromise reducing obtainable trunk blood volume—had that mechanism operated, the loss would have fallen on the haloperidol groups, and it did not. One plausible account of the observed direction is procedural rather than physiological: cataleptic, hypothermic animals are handled with less struggle at decapitation, which may have yielded more consistent trunk blood collection than in the alert control and ILE animals. This remains speculation, and randomisation of processing order would be required to settle it.

The residual concern is loss of power rather than systematic bias: with n = 7–8, the control and ILE groups are the least precisely estimated, which matters most for the oxidative stress comparisons, where the study was already underpowered for the observed effect sizes (Section 4.4), and for the TAS reduction in the ILE group, which rests on seven animals. Selective loss of animals with extreme values within these two groups cannot be formally excluded, since the reasons for loss were technical and recorded only in aggregate.

Sixth, only a single ILE dose and administration schedule was tested. Given the endpoint-dependence of optimal dosing reported by Moshiri et al. [18], dose–response studies are essential before any conclusion about maximal achievable protection. The formulation used (Clinoleic, 80% olive oil/20% soybean oil) is oleic-acid-rich and differs from the soybean-oil-dominant preparations used in most published work; olive-oil-based emulsions contain fewer polyunsaturated fatty acids and may have different pharmacokinetic and pro-oxidant properties [34].

Seventh, mechanistic molecular endpoints—malondialdehyde, reduced glutathione, inflammatory cytokines such as TNF-α and IL-6, and markers of apoptosis—were not measured, so no definitive mechanistic conclusion can be drawn. Tissue haloperidol concentrations were not determined, so the lipid-sink hypothesis could not be tested directly.

Eighth, inter-observer reliability was assessed on a 30% subsample rather than the full cohort. Agreement was high (median weighted κ = 0.893; 95.6% exact agreement; no disagreement exceeding one grade), but the resulting confidence intervals are wide, and reliability in the remaining 70% of slides is inferred rather than measured.

Ninth, humane endpoints were not formally defined in the approved protocol before the study began, and potential confounders were not controlled: cage position on the rack was not randomised, and the order of dosing, sampling and processing was neither randomised nor held constant. In the event no animal reached a state requiring early euthanasia and no mortality occurred, the absence of pre-specified endpoints had no consequence for animal welfare in this study; it is nonetheless a reporting and design shortcoming that should be corrected in future work. The uncontrolled processing order is more consequential, because it prevents the observed pattern of sample attrition from being attributed with confidence to any particular mechanism (Section 4.7, fourth limitation).

Tenth, only male animals were studied, and the findings cannot be generalised to females.

Eleventh, and of particular importance for the veterinary reader, this is a rodent model. Species differences in butyrophenone pharmacokinetics, hepatic metabolism, lipid clearance and dopaminergic receptor distribution mean that neither the injury pattern nor the ILE response described here can be assumed to transfer directly to dogs, cats or wild ungulates. Confirmation in a target species, ideally with tissue drug concentrations and the ILE protocols actually used in small-animal practice, is required before any clinical inference is drawn.

## 5. Conclusions

A single high oral dose of haloperidol (17.5 mg/kg) produced reproducible multi-organ injury in male Wistar rats within 24 h, involving the liver, kidney, brain and testis, with milder cardiac involvement and no pulmonary or ocular effect. The renal finding was the most robust, supported by a large elevation in serum urea with preserved creatinine and by strong correlations between renal histopathology and urea concentration.

Co-administration of intravenous lipid emulsion significantly attenuated microscopic injury in eight of the twelve affected parameters across four organ systems, with no detectable attenuation in the remaining four, with medium-to-large effect sizes, and the testicular spermatogenic endpoint showed the largest improvement, although a residual deficit relative to control cannot be excluded. However, this protection was structurally selective and functionally incomplete: serum urea remained elevated despite improved renal histology, and cardiac findings were unaffected. Lipid emulsion administered alone caused no detectable histopathological change.

Two negative findings carry independent weight. Hepatic transaminases did not rise despite unequivocal hepatic pathology and showed no correlation with it, so normal liver enzymes should not be taken to exclude hepatic injury after haloperidol overdose. Serum oxidative stress indices did not track organ injury, and should be reported precisely: TOS and OSI showed no detectable group differences and no correlation with any injury score, whereas TAS was significantly reduced in the ILE-alone group. The biological meaning of the TAS finding is uncertain, because interference from residual lipaemia with the spectrophotometric assay was not excluded and would also affect the derived OSI. Given this analytical uncertainty and the limited sample size, these indices are best regarded as having provided no usable surrogate information in this model, rather than as having been shown definitively to fail.

Taken together, and within the boundaries of an acute single-dose rodent overdose model assessed at 24 h with an empirical ILE regimen, these data indicate that lipid rescue in butyrophenone toxicity is associated with a differential and structurally incomplete histopathological response among organs at 24 h.

Three observations warrant follow-up in veterinary research, and none should be translated into practice on the strength of these data. Lipid emulsion administered alone produced no detectable histopathological injury in seven organ systems under the specific dose, formulation, species and time point studied, which is a useful but partial safety observation that does not address hypertriglyceridaemia, pancreatitis, fat overload or delayed effects, and which does not constitute evidence that lipid emulsion is safe in any veterinary species. Improvement, where observed, was hepatic, renal, cerebral and gonadal, and the mild histological cardiac changes were unaltered. The cardiac conclusion is limited to the fact that no functional, haemodynamic or electrophysiological endpoint was assessed, so cardiac benefit is untested rather than excluded, and no inference about cardiovascular rescue should be drawn in either direction. And the serum markers used routinely to monitor poisoned animals, namely transaminases and oxidative stress indices, did not reflect the tissue injury present in the same animals at 24 h, so the hypothesis that a reassuring biochemistry panel may not exclude organ injury deserves testing in a target species.

None of these findings should be applied to animal patients. Confirmation in a target veterinary species, using the ILE protocols employed in small-animal practice and incorporating dose-ranging studies, functional and electrophysiological cardiac endpoints, tissue drug and oxidative markers, and endpoints beyond 24 h, is required first.

## Figures and Tables

**Table 1:** Analysable sample sizes by endpoint and group, with reasons for loss.

Group	Histopathology	Routine Biochemistry	Oxidative Stress	Samples Not Obtained
Control	10/10	8/10	7/10	2 insufficient trunk blood volume; 1 clotted before separation
HP	10/10	10/10	10/10	none
HP + ILE	10/10	10/10	10/10	none
ILE	10/10	7/10	7/10	2 insufficient trunk blood volume; 1 clotted before separation
Total	40/40	35/40	34/40	

**Table 2:** Serum oxidative stress parameters. Values are mean ± SD (95% confidence interval).

Parameter	Control (n = 7)	HP (n = 10)	HP + ILE (n = 10)	ILE (n = 7)	*p* ^1^
TOS (µmol H_2_O_2_ Eq/L)	2.27 ± 0.92 (1.43–3.12)	2.39 ± 0.67 (1.91–2.87)	2.72 ± 1.53 (1.63–3.81)	2.00 ± 0.35 (1.67–2.33)	0.431
TAS (mmol Trolox Eq/L)	1.47 ± 0.18 (1.30–1.64)	1.34 ± 0.37 (1.08–1.61)	1.28 ± 0.16 (1.17–1.40)	1.13 ± 0.16 ^2^ (0.99–1.28)	0.028
OSI (arbitrary unit)	0.154 ± 0.054 (0.104–0.205)	0.191 ± 0.067 (0.142–0.239)	0.213 ± 0.109 (0.135–0.291)	0.180 ± 0.041 (0.142–0.218)	0.512

^1^ Kruskal–Wallis omnibus test. ^2^ *p* = 0.011 vs. control (Mann–Whitney U). TOS, total oxidant status; TAS, total antioxidant status; OSI, oxidative stress index; HP, haloperidol; ILE, intravenous lipid emulsion.

**Table 3:** Serum biochemical parameters. Values are mean ± SD (95% confidence interval).

Parameter	Control (n = 8)	HP (n = 10)	HP + ILE (n = 10)	ILE (n = 7)	*p* ^1^
AST (IU/L)	131.3 ± 30.7 (105.6–157.0)	117.1 ± 34.8 (92.2–142.0)	142.6 ± 36.9 (116.2–169.0)	133.8 ± 35.3 (101.1–166.4)	0.488
ALT (IU/L)	54.7 ± 14.8 (42.3–67.1)	43.5 ± 18.2 ^2^ (30.5–56.5)	59.5 ± 35.3 (34.2–84.7)	45.9 ± 8.7 (37.9–54.0)	0.220
ALP (IU/L)	213.2 ± 33.4 (185.2–241.1)	178.0 ± 44.9 (145.9–210.2)	170.8 ± 27.4 ^2^ (151.2–190.4)	194.5 ± 40.5 (157.1–231.9)	0.097
LDH (IU/L) ^3^	745.2 ± 245.9 (n = 7)	708.1 ± 277.6 (n = 10)	802.2 ± 181.9 (n = 7)	549.1 ± 373.7 (n = 7)	0.463
Urea (mg/dL)	46.5 ± 8.8 (39.1–53.9)	67.4 ± 13.2 ^4^ (58.0–76.9)	61.5 ± 8.7 ^4^ (55.3–67.7)	41.9 ± 3.5 (38.7–45.1)	<0.001
Creatinine (mg/dL)	0.261 ± 0.038 (0.230–0.293)	0.287 ± 0.039 (0.259–0.315)	0.283 ± 0.022 (0.267–0.299)	0.254 ± 0.041 (0.217–0.292)	0.205
CK-MB (IU/L)	514.0 ± 188.8 (356.2–671.8)	418.0 ± 129.9 (325.0–510.9)	575.7 ± 212.7 (423.6–727.9)	344.5 ± 198.3 (161.0–527.9)	0.158

^1^ Kruskal–Wallis omnibus test. ^2^ *p* < 0.05 vs. control (Mann–Whitney U)—note that both differences are in the direction of *lower* enzyme activity. ^3^ Four haemolysed samples excluded (see Section 2.6). ^4^ *p* ≤ 0.003 vs. control. AST, aspartate aminotransferase; ALT, alanine aminotransferase; ALP, alkaline phosphatase; LDH, lactate dehydrogenase; CK-MB, creatine kinase-MB.

**Table 4:** Sensitivity of biochemical group comparisons to the handling of the four haemolysis-flagged samples. Values are Kruskal–Wallis *p*.

Analyte	All Samples Retained	Flagged Samples Excluded	90th-Percentile Winsorisation	Rank Transformation
LDH	0.109	0.463	0.110	0.103
AST	0.488	0.608	0.451	0.506
CK-MB	0.158	0.560	0.159	0.157
ALT	0.220	0.235	0.216	0.223
ALP	0.097	0.092	0.098	0.091
Urea	<0.001	<0.001	<0.001	<0.001

**Table 5:** Semi-quantitative histopathological scores across seven organ systems (n = 10 per group). Values are median (minimum–maximum).

Organ	Parameter	Control	HP	HP + ILE	ILE	*p* ^1^	*q* ^2^
Liver	Inflammation	0 (0–0)	2 (1–3)	1 (0–2)	0 (0–0)	<0.001	<0.001
	Stromal expansion	0 (0–0)	1.5 (1–3)	1 (0–2)	0 (0–0)	<0.001	<0.001
	Congestion	0 (0–0)	2 (1–3)	1 (0–2)	0 (0–0)	<0.001	<0.001
	Degeneration	0 (0–0)	2 (1–3)	1 (0–2)	0 (0–0)	<0.001	<0.001
Lung	Inflammation	0 (0–0)	0 (0–1)	0 (0–0)	0 (0–0)	0.104	0.119
	Stromal expansion	0 (0–0)	0 (0–1)	0 (0–0)	0 (0–0)	0.392	0.392
	Congestion	0 (0–0)	0 (0–1)	0 (0–0)	0 (0–0)	0.104	0.119
Kidney	Inflammation	0 (0–0)	1.5 (1–2)	1 (0–1)	0 (0–0)	<0.001	<0.001
	Stromal expansion	0 (0–0)	1 (1–2)	1 (0–1)	0 (0–0)	<0.001	<0.001
	Congestion	0 (0–0)	1.5 (1–2)	1 (0–1)	0 (0–0)	<0.001	<0.001
Testis	Johnsen score ^3^	8 (7–8)	6 (6–7)	7 (6–8)	8 (7–8)	<0.001	<0.001
Heart	Congestion	0 (0–0)	0 (0–1)	0 (0–1)	0 (0–0)	0.035	0.047
	Degeneration	0 (0–0)	0.5 (0–1)	0 (0–1)	0 (0–0)	0.012	0.017
Brain	Oedema	0 (0–2)	2 (1–2)	1 (0–2)	0 (0–0)	<0.001	<0.001
	Inflammation	0 (0–2)	1.5 (1–2)	1 (0–2)	0 (0–0)	<0.001	<0.001
Eye	Degeneration	0 (0–0)	0 (0–1)	0 (0–0)	0 (0–0)	0.392	0.392
	Congestion	0 (0–0)	0 (0–0)	0 (0–0)	0 (0–0)	n.e. ^4^	n.e.

^1^ Kruskal–Wallis omnibus test. ^2^ Benjamini–Hochberg FDR-adjusted *p*-value across the 16 evaluable parameters. ^3^ Modified Johnsen score (1–10); higher values indicate more complete spermatogenesis. ^4^ n.e., not evaluable (zero variance across all groups). HP, haloperidol 17.5 mg/kg; ILE, intravenous lipid emulsion 7.5 mL/kg × 2.

**Table 6:** Pairwise comparisons for parameters with a significant omnibus effect (Mann–Whitney U test, two-sided; n = 10 per group).

Organ	Parameter	Control vs. HP *p* (*q*)	HP vs. HP + ILE *p* (*q*)	*r* ^1^	Control vs. HP + ILE *p*
Liver	Inflammation	<0.001 (<0.001)	0.019 (0.038)	0.55	<0.001
	Stromal expansion	<0.001 (<0.001)	0.031 (0.053)	0.51	<0.001
	Congestion	<0.001 (<0.001)	0.007 (0.032)	0.64	<0.001
	Degeneration	<0.001 (<0.001)	0.015 (0.035)	0.59	<0.001
Kidney	Inflammation	<0.001 (<0.001)	0.006 (0.032)	0.65	0.002
	Stromal expansion	<0.001 (<0.001)	0.011 (0.035)	0.58	0.002
	Congestion	<0.001 (<0.001)	0.004 (0.032)	0.70	0.005
Testis	Johnsen score	<0.001 (<0.001)	0.008 (0.032)	0.66	0.072 (n.s.)
Brain	Edema	<0.001 (<0.001)	0.015 (0.035)	0.58	0.008
	Inflammation	<0.001 (0.001)	0.033 (0.053)	0.50	0.008
Heart	Degeneration	0.014 (0.020)	0.398 (0.424)	0.20	0.077 (n.s.)
	Congestion	0.034 (0.045)	0.681 (0.681)	0.10	0.077 (n.s.)

^1^ Rank-biserial correlation for the HP vs. HP+ILE comparison. *q*, Benjamini–Hochberg adjusted *p*-value within each comparison family. n.s., not significant.

**Table 7:** Inter-observer agreement between two independent blinded pathologists on a stratified random 30% subsample (12 animals; 203 paired observations).

Organ	Parameter	Paired n	Exact Agreement	Within ±1 Grade	Weighted κ ^1^	95% CI ^2^
Liver	Inflammation	12	91.7%	100%	0.872	0.52–1.00
	Stromal expansion	12	91.7%	100%	0.857	0.63–1.00
	Congestion	12	83.3%	100%	0.786	0.49–1.00
	Degeneration	12	91.7%	100%	0.885	0.65–1.00
Lung	Inflammation	12	91.7%	100%	0.625	0.00–1.00
	Stromal expansion	12	100%	100%	n.est. ^3^	—
	Congestion	12	100%	100%	n.est.	—
Kidney	Inflammation	12	91.7%	100%	0.898	0.67–1.00
	Stromal expansion	12	100%	100%	1.000	1.00–1.00
	Congestion	12	91.7%	100%	0.893	0.58–1.00
Testis	Johnsen score ^4^	11	100%	100%	1.000	1.00–1.00
Heart	Congestion	12	91.7%	100%	0.818	0.60–1.00
	Degeneration	12	100%	100%	1.000	1.00–1.00
Brain	Edema	12	100%	100%	1.000	1.00–1.00
	Inflammation	12	100%	100%	1.000	1.00–1.00
Eye	Degeneration	12	100%	100%	n.est.	—
	Congestion	12	100%	100%	n.est.	—

^1^ Linear weights for 0–3 ordinal parameters; quadratic weights for the 1–10 Johnsen score, both pre-specified. ^2^ Bootstrap percentile interval, 5000 replicates. ^3^ n.est., not estimable: both observers assigned a single invariant category, so kappa is undefined despite complete agreement. ^4^ One animal is excluded because the Johnsen score was not recorded for that slide by both readers.

**Table 8:** Spearman rank correlations between histopathological scores and biochemical parameters.

Histopathological Score	Biochemical Parameter	n	ρ	*p*
Renal inflammation	Urea	35	+0.735	<0.001
Renal congestion	Urea	35	+0.689	<0.001
Renal stromal expansion	Urea	35	+0.597	<0.001
Testicular Johnsen score	Urea	35	−0.451	0.007
Hepatic inflammation	ALP	35	−0.342	0.044
Hepatic congestion	AST	35	−0.165	0.344
Hepatic inflammation	ALT	35	−0.159	0.362
Hepatic degeneration	AST	35	−0.097	0.578
Hepatic stromal expansion	ALT	35	−0.022	0.902
Cerebral oedema	OSI	34	+0.018	0.920
Cerebral inflammation	OSI	34	+0.008	0.963
Testicular Johnsen score	OSI	34	−0.040	0.821

## Data Availability

The datasets generated and analysed during the current study are provided in full as Supplementary Tables S1–S3 and are available from the corresponding author upon reasonable request. The archived histological sections and paraffin blocks are no longer available (Section 2.11), so the biological specimens underlying the histopathological scores cannot be shared or re-examined.

## Supplementary Materials

The following supporting information can be downloaded at: https://www.mdpi.com/article/10.3390/vetsci13090890/s1, Table S1: Individual animal histopathological scores across seven organ systems. Table S2a: Individual animal biochemical values. Table S2b: Individual animal oxidative stress values. Table S3a: Histopathological scores assigned by Pathologist 1 in the inter-observer reliability subsample. Table S3b: Histopathological scores assigned by Pathologist 2 in the inter-observer reliability subsample. Table S3c: Weighted kappa statistics for inter-observer reliability across histopathological parameters.

## Abbreviations

ALP, alkaline phosphatase; ALT, alanine aminotransferase; ANOVA, analysis of variance; AST, aspartate aminotransferase; CK-MB, creatine kinase-MB; FDR, false discovery rate; H&E, haematoxylin and eosin; HP, haloperidol; ILE, intravenous lipid emulsion; LAST, local anaesthetic systemic toxicity; LDH, lactate dehydrogenase; OSI, oxidative stress index; ROS, reactive oxygen species; TAS, total antioxidant status; TOS, total oxidant status.
